# Between God and Nation: The Colonial Origins of Democracy Support in British Africa

**DOI:** 10.1007/s12116-024-09450-2

**Published:** 2024-12-06

**Authors:** Bastian Becker, Dean Dulay

**Affiliations:** 1 Humboldt-University of Berlin, Berlin, Germany; 2 Singapore Management University, Singapore, Singapore

**Keywords:** Africa, Development, Democracy, Education, Religion

## Abstract

Popular support is important to the emergence and survival of democracy. In this paper, we study how church-state relations in former British Africa shaped long-run support for democracy. We argue that shared national identity was the basis for incentive alignment, facilitating cooperation over educational policy between church and state and engendering long-run support for democracy. Employing geospatial analyses of historical mission data and contemporary social survey data from 19 former British African colonies, we find that areas with missions originating from Britain exhibit better educational outcomes and greater support for democracy than areas that had non-British missions. Furthermore, areas with British Protestant missions show greater support for democracy than British Catholic missions, suggesting that Protestantism supplements the benefits of shared nationhood between mission and state. Our findings add nuance to scholarship on the colonial origins of democracy by highlighting the interdependent legacies of church and state.

**Supplementary Information:**

The online version contains supplementary material available at 10.1007/s12116-024-09450-2.

## Introduction

British colonial rule is associated with relatively benign economic and political legacies (Acemoglu et al. 2001; Lee and Paine 2019; Frankema 2012), since former British colonies tend to be richer and more democratic than other postcolonial states. Scholars posit that a major factor that set the British apart was their reliance on Protestant missions in providing formal education, and indeed, a large literature has established that Protestant missions advanced human capital and overall development among native populations across the colonial world (Woodberry 2012; Nunn 2014; Becker 2021; Wantchekon et al. 2015). Extant work therefore attests to Protestantism as a major catalyst behind why British colonialism led to relatively good developmental outcomes (Lankina and Getachew 2013, 2012; Gallego and Woodberry 2010).

The literature implicitly presumes a homogeneous relationship between the British colonial state and Protestant missions: all Protestant missions are “the same,” and the colonial state treated them in the same way. The assumption of a simple British Protestant binary masks considerable variation of the ways in which Protestant missions differed in their relationship with the British colonial state, and how this variation shaped the contours of economic and political development (Becker and Schmitt 2023). This assertion seems plausible, since colonial missions varied across several dimensions, among which are the national identity of the mission itself—in our case, whether the mission originated from Britain or from some other colonial power—and whether they share the same national identity as the colonial state. Yet a large literature on shared identity underscores its evident importance for fostering trust, facilitating communication, and exchange, factors that necessarily underpin the relationship between the British colonial metropole and the Protestant missions on the ground (Tanis and Postmes 2005; Foddy et al. 2009; Voci 2006).

In this paper, we argue that shared national identity between British Protestant missions and British colonial governments lowered transaction costs for both, leading British governments to direct support disproportionately to British missions, thus allowing these well-supported missions to increase human capital in ways that increased long-run support for democracy. Our argument is grounded on the following logic: British governments and Protestant missions shared a strong interest in promoting education, albeit for different reasons. These aligned interests opened up space for mission-state cooperation. The colonial government would provide resources to the mission, while the missions provided education in lieu of the state. However, the colonial state was limited both in its resources and capacity to monitor and enforce the missions and thus had to be selective about which missions it chose to support. Shared national identity was a decisive factor in determining support. Colonial governments preferred collaborating with missions originating in their home country that represented the dominant religious denomination(s) of the metropolitan state, as this engendered trust, and eased communication and coordination. As a result, British Protestant missions received greater governmental support to expand education than non-British Protestant missions, which promoted both human capital and democratic norms. These effects persist into the present, such that individuals in areas that had British Protestant missions are more educated and show greater support for democracy than in areas that did not.

We examine our argument by employing geospatial analyses of historical mission data and social survey data from 19 former British African colonies. Drawing on the World Missionary Atlas (Beach and Fahs 1925), we identify the precise location of 1895 Protestant missions and their country of origin.^1^ Our contemporary data on educational outcomes and support for democracy comes from the geocoded sixth wave of the Afrobarometer survey. Our main analyses focus on 48,250 respondents in 19 former British colonies.^2^ We estimate a series of linear regression models that account for spatial clustering to test our hypotheses, while also including an extensive set of controls to account for the non-randomness of mission settlement (Jedwab et al. 2022), including precolonial variables capturing early economic and political development as well as geographic and climatic conditions, and colonial variables capturing colonial infrastructure, distances from colonial centers, economic activities such as mining and cash cropping, and the presence of Catholic missions as a form of religious competition.

Consistent with our argument, we find a stronger relationship between British Protestant missions and long-run support for democracy than for non-British missions. We also find few differences between locations with Non-British missions and locations without any missions, which suggests that shared national origins were necessary for missions to affect long-run support for democracy. Furthermore, we show that education is a primary mechanism by which these contrasting effects accrue: British mission areas have higher levels of primary education than non-British mission areas. The results are robust to a variety of sensitivity tests, such as the use of alternative measures of (British and non-British) mission settlement and the education variable, the removal of potential outlier countries, and alternative clustering of standard errors. They also hold when limiting our analyses to precolonial missions, which avoids potential self-selection biases. Finally, we explicitly test the scope conditions of our argument and find that national origins are specific to Protestant missions in British Africa. We find that British missions in French Africa, and Catholic Missions in British Africa as well as in French Africa, are not associated with higher levels of contemporary support for democracy.

Our paper makes three important contributions. First, we add to extant literature that examines the effects of the Christian missions in Africa (Okoye 2021; Becker 2021; Woodberry 2012; Nunn 2014; Lankina and Getachew 2013; Wietzke 2015; Ananyev and Poyker 2021; Cagé and Rueda 2020) and beyond (Valencia Caicedo 2019; Dulay 2022; Calvi et al. 2020; Lankina and Getachew 2012). It is not disputed that the colonial missions touched almost all aspects of colonial life. This paper builds on previous work that has already examined other social, economic, and political impacts of missionary settlement, both in the immediate past and their ramifications in the present. Specifically, our work adds another element to existing work that examines the causes and consequences of support for democracy in contemporary Africa (Bassett and Clarke 2008; Bratton and Mattes 2001; Evans and Rose 2007). This paper builds on these literatures by further unpacking the relations between colonial missions and the colonial state by examining how shared national identity shaped the dynamics of their interactions.

Second, we contribute to emerging scholarship on the role of national networks in colonial empires. While such networks have been studied extensively by historians, social scientists have only recently begun testing arguments with modern analytical methods (Xu 2018). Becker and Schmitt (2023) show that national ties between British governments and missions already led to better educational outcomes in the colonial era. Furthermore, French governments restricted missionary activity heavily, making it especially hard for non-French missions to enter (Becker 2022). This governmental pre-screening and the selection of “friendly” missions might explain why we find national origins to make no difference in French colonies. Our paper demonstrates that national alignment in the colonial era, in particular between missions and states, continues to shape development in the present.

Third, we add to literature that highlights the state-building role of Christian missions. As such, missions have been construed as extensions of the state (Amasyalı 2021; Dulay 2022; Gill 2008; Okpalike and Nwadialor 2015). Our findings suggest an alternative means by which missions contributed to state-building in British Africa: the unintended spread of democratic norms, particularly in places where missions had strong ties with colonial governments. In this case, missions contributed not just to building up democratic institutions but also to fostering underlying support for democracy that makes these institutions robust.

The paper proceeds as follows. The second section provides a highly condensed summary of the historical literature and develops our theoretical arguments. The third section presents our data and research design. The fourth section presents our main results, while the fifth section examines the scope conditions of our argument. The sixth section offers concluding remarks.

## Theory

Much has been made of the positive effects of British colonialism on democracy and of the role of colonial missions in facilitating long-run human capital improvements. More recent work has explicitly linked these two strands of literature, arguing that British colonialism’s effect on democracy was facilitated by the mass education programs of colonial missions (Lankina and Getachew 2012, 2013; Gallego and Woodberry 2010). This research program has significantly advanced our understanding of colonialism’s democratic impacts and the role colonial missions played in it. Yet colonial missions vary in significant ways. For example, Protestant missions have been shown to be particularly beneficial for long-run democratization (Woodberry 2012).^3^ But colonial missions also varied in terms of national identity. For example, British missions may have been favored by the British colonial state. The confluence of religious denomination and national identity was omnipresent in the scramble for Africa, as religious competition between Protestants, Catholics, and even Muslims, and competition between colonial powers such as Britain, France, and Germany over territory intersected in myriad ways as these various groups sought evangelization and exploitation throughout the continent. How did this mix of religious denomination and national identity coalesce with the goals of colonial missions and colonial states to affect democratization?

We argue that a particular combination of religious denomination and national identity were contributing factors for improving long-run democratization. The British colonial state cooperated with British Protestant missions to spread education throughout colonial Africa. This increase in education in turn led to the embedding of democratic values that has persisted into the present. But both the national identity of the colonial mission in relation to the British state, as well as the religious denomination of the mission, significantly moderate this process.^4^ In particular, support for democracy is higher in areas that had British Protestant missions versus areas with British Catholic missions or non-British Protestant missions, suggesting that the confluence of shared British identity between mission and state, and the particulars of Protestantism are contributing factors for the higher levels of contemporary support for democracy. In the case of colonial British Africa, a mission needed to be both Protestant and British for it to bring about long-run support for democracy.

The argument is grounded on key presumptions. First, while the colonial state and colonial missions were sometimes at odds, they shared political goals that induced cooperation between them. Second, national identity lubricated cooperation between British missions and the British state by facilitating trust between them, a product of shared norms and the exigencies of the competition between European states in Africa. Finally, education positively correlates with support for democracy, and moreover, Protestant missions facilitated education-led democratization in a way Catholicism did not, leading to higher levels of support for democracy in British Protestant versus British Catholic missions. The following discussion expounds on these presumptions.

### The Shared Grounds for Cooperation Between Church and State

While the colonial state and colonial mission were sometimes at odds, both sides faced clear incentives for collaboration. Christian missions wanted to evangelize the local population, usually through schooling and education, while colonial administrators wanted to increase human capital as a means of developing the colonial economy. The implication is that while there were differences in how the colonial state was managed, both Church and state tacitly agreed that some degree of control was important. Since both Church and state had incentives to increase human capital they had an incentive to cooperate. This underlying incentive compatibility was also true in a colonial African context. Hastings (1994, p.413) notes the following:

[Missionaries] shared in the expansionist imperialism of the age, an easy belief that within the providence of history Africa had now to be conquered for its own good. Their immediate concern was that it should be conquered in a humane way and by the right power.

The codependence of the mission and the state is evident in colonial Africa. Moreover, British colonialism stands out as particularly reliant on missions for educating local populations. The state understood the importance of education, considering it “indispensable to the functioning of (…) [colonial] administration and of the commercial houses, for they could not afford to employ whites in subaltern posts” (Crowder 1968, p.369). But the state faced budgetary constraints and thus faced incentives to outsource the task of educating the native population. The colonial government was therefore willing “to leave the bulk of the primary education to the missionary bodies” and aimed at expanding the reach of mission education through grants-in-aid and land allocations (Buell 1928, p.728). Grants in-aid were used to build schools, to pay teacher salaries, and to buy equipment and furniture. On the other hand, missions also had limited financial resources and relied on the colonial state to expand educational services. The Ugandan Education Report of 1929 states that missions “must be given the sole credit for educational development in the country.” It also underscores missions’ reliance on government largesse: “But the missions had neither the staff nor the money needed, nor could they wield the necessary authority. Without the ordered government, the communications, the friendly support of the administration, the missions could never have developed their work as they did.”

### National Identity Facilitated Cooperation

While incentive compatibility formed the basis of cooperation between the colonial church and colonial state, shared national identity determined which missions the colonial state chose to align with in practice. Colonial states in Africa faced limited resources and made rational calculations regarding the costs and benefits of expansion (Herbst 2000). As such, they sought to minimize transaction costs when engaging in relations with colonial missions. A common national identity reduces transaction costs in subtle but important ways. Shared national identity between the British colonial state and British missions thus facilitated cooperation between the two. The colonial state therefore prioritized sending resources and support to British missions as opposed to non-British ones.

Sharing a common national identity facilitates trust between the mission and the state. A large theoretical literature has explicitly made a link between higher trust, lower transaction costs, and more mutually beneficial exchange (Bromiley and Harris 2006; Hill 1990; Greif 1993; Williamson 1979). Trust is important for mutually beneficial exchange because both parties in an exchange must believe that the other party will not renege on their agreement in case standard monitoring and enforcement mechanisms are insufficient. Literature in social psychology has also established that trust is a product of relational and socio-cultural mechanisms, as shared identity lubricates social relations and engenders trust as a product of the real and presumed similarities between members of in-groups (Foddy et al. 2009; Voci 2006). The converse is also true: lower trust in turn increases transaction costs and prevents otherwise mutual beneficial cooperation from taking place. In the context of our argument, trust between mission and state allows them to engage in policies that are mutually beneficial to both sides.

Shared national identity, in turn, facilitates trust as it fosters both its shared relational and socio-cultural benefits, as well as the ease of monitoring and enforcement. We argue that shared norms and beliefs between the British mission and the British state, the state’s ability to monitor and enforce mission behavior, and the resources the mission received from the state lowered transaction costs enough for both to engage in cooperative behavior. There is significant historical evidence to support this assertion. This is most evident in how the British colonial government treated non-British missions. One example is from Tanganyika, a former British colonies. Once the British colonized the country they removed over 400 German missions because the governor-general Sir Horace Byatt refused to cooperate and provide resources to these German missions. They were eventually replaced by British missions (Phelps-Stokes Fund 1922; Buell 1928). Colonial governments were generally distrustful of foreign (i.e., non-British) missions, fearing subversive behavior that would undermine British interests. Colonial missions also played into inter-governmental antagonisms. A Protestant missionary in Nigeria complained: “I saw at Lokoja the French tricolour flag flying above which is still called the French Mission and not the Catholic Mission” (Ekechi 1972, p.86).

British missions were also favored by the British colonial government because of ease of enforcement. They benefited not only from their close relationships to the colonial government in the colonies but also to the Colonial office in London (Whitehead 2007). This cooperation in the form of frequent consultations (official and unofficial) between British mission societies and British state and especially collaborations in committees was “most heartily appreciated and highly valued” by both sides. Further evidence of this close collaboration was the fact that British mission societies were also represented on the Advisory Committee on Colonial Education in Tropical Africa, a de facto executive body under the Colonial Secretary that was imperative for controlling education in British Africa (D’Souza 1975). The committee published and adopted reports, memoranda, and directives, which underscored the importance of collaboration between missions and governments in the provision of educational services. Finally, alignment works both ways: British Protestant missions were able to lobby the British government for additional funding for education to the colonies in the 1920 s (Beck 1966). In summary, shared national identity fostered trust between British colonial state and British missions, and this trust underpinned the close collaboration and selective funneling of resources between them, above and beyond what was provided to missions that did not share British identity.

### The Link Between Education, Protestantism, and Democracy

The argument that education led to long-run increases in support for democracy is buttressed by a large literature arguing for the positive link between education and support for democracy.^5^ Cross-country empirical work shows that the more educated countries are, on average, the more democratic they tend to be (Barro 1999, S6). Theoretical explanations are also abundant. In his classic text, Lipset (1959, p. 79) makes a norm-based argument for how education affects democracy, claiming that education “broadens men’s outlooks, enables them to understand the need for norms of tolerance, restrains them from adhering to extremist and monistic doctrinies, and increases their capacity to make rational electoral choices.” Relatedly, Glaeser et al. (2007) argue that schooling teaches people to interact with others and raises the benefits of civic participation, including voting and organizing, both fundamental activities of democratic governance.

These broad arguments are further supported by research that explicitly links missions to educational attainment. The research posits literacy in particular to be an important factor. Mission presence meant increasing literacy as a means towards religious indoctrination. Literacy in turn served as catalyst for a variety of mechanisms that spurred democratization. First, literacy facilitated economic development. Richer countries tend to be more democratic. Moreover, mission-driven literacy promoted social inclusivity and led to social reform movements (Lankina and Getachew 2021). Finally, a large body of empirical research has provided empirical support for the link between mission presence and human capital accumulation, finding a positive association between mission presence and education in contexts as diverse as India, Argentina, and the Philippines (Lankina and Getachew 2012; Valencia Caicedo 2019; Dulay 2022).

Moreover, the processes by which education (literacy) led to democratization were significantly amplified by Protestant missions, over and above missions from other religious groups. Woodberry and Shah (2004) argue that Protestant missions were the progenitors of many of the organizational forms that spurred social inclusion and collective mobilization. In addition, Protestants promoted literacy in line with the *sola scriptura*, which advocated for individual bible study in the pursuit of religious faith. This is supported by empirical work that finds a positive association between Protestant missions and female education (Becker and Woessmann 2009; Nunn 2014). Indeed, Calvi et al. (2022) assert that in India Protestant missions were in fact the pioneers of female education, as colonial authorities were reluctant to take up the issue of female education, fearing hostility from locals.^6^ Protestant missions also advocated for mass literacy in a way that other religions denominations did not. Protestant missions pushed mass literacy (as part of a broader educational package) so converts could read and interpret the Bible, which they believed as necessary to convert the native population. The increasing circulation of Western texts as well as novel anticolonial and antiracist scholarship exposed the newly literate to a broad spectrum of liberal ideas (Boahen 1989).

The claim that literacy, as an integral component of Protestant missions’ educational efforts, planted the seeds of nascent democratization is also explicitly supported by historians of Protestantism in Africa. An explicit link between mass education and democratization has been posited by eminent historians of colonial Africa. Porter (2004, p.318) states the following:

[...] missionary work and education, despite their manifest limits, often had a vital liberating impact and was welcomed for that reason. There is no doubt that the spread of literacy and knowledge of other languages both widened horizons at many different social levels and greatly enhanced the ability of ordinary people to question or subvert traditional attitudes as well as imperial and colonial assumptions.

There is also extant historical research that argues for education’s influence towards democratization in Africa. First, education built a nascent elite African class that eventually pushed for democratization. Within the bundle of skills that education provides, literacy has been argued for as necessary for the “enlightenment” of an elite class of young nationalists. Windel (2009, p.167) makes this view explicit when he says:

[...] the nucleus of a modern, educated African elite that would one day be the vanguard of national liberation struggles in the mission schoolrooms where they read in English and were thus trained in careers that put them at a distinct advantage as Africa underwent drastic changes in economy and the structure of society during the first half of the twentieth century.

In summary, British Protestant missions were able to more widely spread schooling and education in the local population than non-British missions and British Catholic missions. This led to a long-run divergence in support for democracy through a combination of nascent elite formation and the teaching of values that buttressed support for democracy. The argument leads us to formulate the following two hypotheses:

- H1 (education mechanism): Historical exposure to British Protestant missions increases citizens’ educational attainment more than exposure to non-British missions.
- H2 (support for democracy): Historical exposure to British Protestant missions increases citizens’ support for democracy more than exposure to non-British missions.

## Research Design

Our analysis combines contemporary survey data with a rich set of historical variables. Our survey data comes from the Afrobarometer project, an independent research network that has been conducting comparative social surveys since 1999 across a growing number of African countries. The nationally representative surveys cover a wide range of socio-economic and political topics. We use data from the two latest geocoded waves of the Afrobarometer survey, which were conducted in 2011–2013 (wave 5) and 2014–2015 (wave 6), respectively (Afrobarometer 2016; BenYishay et al. 2017). The use of two surveys gives us broader geographic coverage within each country and allows us to account for survey-specific effects.

Most historical data stems from digitized maps, which allows us to capture the colonial and precolonial situation in each survey location. Variables include local conditions, such as the presence of a colonial mission, our main independent variable, as well as distances to important locations, such as the coastline. Overall, our data includes 56,547 survey respondents from 19 former British colonies in Africa.^7^

We analyze the data using linear and logit models with robust standard errors clustered by survey location.^8^ In most of our models, we capture exposure to Protestant missions through the inclusion of two variables, one for British missions and the other for non-British missions. This accounts for any confounding effects that might arise from correlational patterns in the locations of British and non-British missions. For the same reason, we control for the presence of Catholic missions. As Catholics—similar to non-British Protestant missions—also invested in schooling but were not closely connected to the British government, the estimated effects are also of substantive interest to us.

Our models include an extensive set of precolonial and colonial control variables to account for potential confounders. In addition, we limit the sample to locations within 100 km of colonial missions to provide more plausible counterfactuals in estimating the effects of mission exposure (Cagé and Rueda 2016; Becker 2021).^9^ We also show that our results are not driven by outlying cases through leave-one-out regressions (see Supplementary Material, Figures A1–A4).

To further probe the causal mechanisms suggested by our argument, we perform two additional analyses. First, we look more closely at the relationship between education and democratic attitudes. To this end, we present regression results of the direct effect of education on support for democracy and conduct a mediation analysis to measure the degree to which the democracy effect is channeled through education. Second, we address endogeneity that might result from the self-selection of missions into different colonies by exploiting the fact that many mission stations were set up before British colonization. We re-estimate our main models but restrict the sample to respondents in the proximity of these early mission stations. The results show that our main findings are not driven by self-selection.

### Data and Measurement

#### Dependent Variables

##### Education

We operationalize our education variable by constructing a dummy for whether the respondent completed *primary education*. Few mission schools offered educational opportunities beyond the primary level, and legacies are therefore best traced by focusing on this level (Becker 2021). That being said, mission legacies should also affect educational attainment beyond primary school. As a more fine-grained measure of the educational legacies and in line with many other studies (Nunn 2014), we also use *years of education* in our analyses. As our individual data is drawn from the Afrobarometer waves 5 and 6, it reflects outcomes between 2011 and 2015.

##### Democratic Attitudes

We use two variables to capture attitudes towards democracy. First, *support for democracy* is a binary variable: Respondents that endorse the statement that “democracy is preferable to any other kind of government” are assigned the value 1, and 0 otherwise. This variable is commonly used as a measure of support for democracy (e.g., Evans and Rose 2012).

Second, we use a continuous variable that focuses more specifically on elections. *Support for elections* indicates whether respondents choose the statement “ We should choose our leaders in this country through regular, open and honest elections” over an alternative statement, “Since elections sometimes produce bad results, we should adopt other methods for choosing this country’s leaders.” Responses are collected on a five-point scale, ranging from full agreement with the second statement to full agreement with the first statement.

**Fig. 1:**
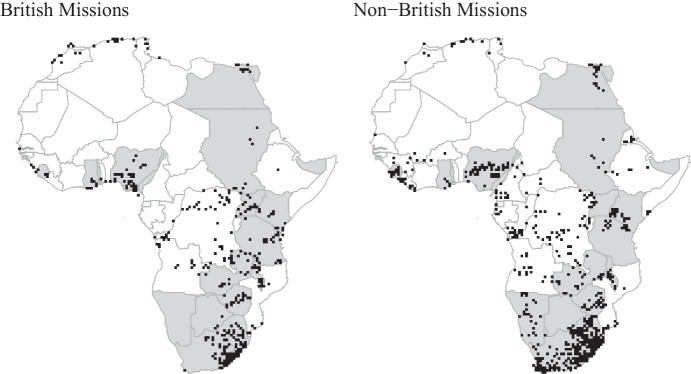
Protestant mission fields in British Africa and beyond

#### Independent Variables

##### Mission Exposure

Our main independent variable is the exposure of localities to British and non-British missions in the colonial era. To construct this variable, we draw on the 1925 *World Missionary Atlas* (WMA) by Beach and Fahs (1925), which includes information on the location and national origin of 1895 Protestant mission stations in Africa (see Fig. 1). The WMA has been digitized and geocoded by Becker (2022).

Two binary variables constitute our main indicators for mission exposure. A locality is coded as exposed to a *British mission (Dummy)*, taking value 1, if it is situated within 25 km of a station that belonged to a British mission society, and 0 otherwise.^10^ Similarly, the second binary variable, *Non-British mission [Dummy]*, indicates whether the locality is within 25 km of a station that was associated with a society from outside of Britain.^11^ Note that some localities are exposed to both kinds of missions, in which case both variables take the value 1. A potential concern is that the results will not hold under alternative radii. In our robustness tests, we examine alternative radii as a measure of mission exposure (see Supplementary Material, Tables A11–A13).

While the use of the binary variable for British and non-British mission settlement is intuitive and easy to interpret, there is important variation along the intensive margin that cannot be captured by this measure. Most important are the number of stations in a given locality as well as the time since the first station was established. Considering the intensive margin is especially important when conceptualizing missions as extensions of the state, because previous scholarship has established an empirical relationship between state longevity—how long a state has been in place—and economic development (Pierskalla et al. 2017). Our first intensive margin measure counts the number of mission stations in a locality, which again is defined as the area within 25 km of a respondent. The two resulting variables *British mission (Count)* and *Non-British mission (Count)* distinguish between the two kinds of missions. We therefore construct an intensive margin measure of mission settlement by determining the duration of mission exposure in each location. The two measures, *British mission (Time)* and *Non-British mission (Time)*, indicate how long ago—in centuries—a British or non-British station had been set up, within a radius of 25 km. In particular, we compute the difference between 1930 and the year of establishment.^12^ For example, if the first mission in a location was set up in 1900, a value of 0.3 is assigned, as the mission was set up 30 years prior. To estimate the intensive margin effect, we only include locations with Protestant mission stations (whether British or non-British) in the corresponding models.

We also show that our results are robust to excluding German colonies, which were taken over by Britain in the aftermath of World War I leading to the large-scale expulsion and replacement of German missionaries (see Supplementary Material, Table A5). The results are also robust to excluding traditionally Muslim areas, which Christian missions were often barred from entering and were unable to attain large numbers of converts in cases where they did (see Supplementary Material, Table A6).

#### Control Variables

Our identification strategy does not employ experimental or quasi-experimental variation. As such, a causal interpretation of the relationship between British and non-British mission settlement on long-run outcomes hinges on the inclusion of relevant control variables. Mission stations were not set up in random locations. The causal association of mission settlement with contemporary political outcomes might therefore be confounded by selection in terms of non-random mission settlement. Furthermore, missionary activity was not independent of other colonial interventions. It is therefore necessary to account for other precolonial and colonial variables that might otherwise confound long-term effects. Finally, we also include a small set of individual variables that do not suffer from post-treatment bias.

In the following, we provide a short overview of all control variables. Supplementary Material A details the rationale behind the inclusion of each variable, explains its operationalization, and lays out the data sources.

##### Precolonial Variables

Missions preferred locations that were easily accessible and had a hospitable environment. We therefore account for a set of geographic factors: distance to the coast, access to navigable waterways, altitude, terrain ruggedness, malaria burden (1900), and agricultural suitability. In addition, we account for social conditions: population density (1900), political centralization of ethnic groups, distance from Muslim centers, slave trade exposure, ethnic belief in high gods, and polygamy.

##### Colonial Variables

Missions were closely entangled with the colonial enterprise at large. Therefore, we account for a broad range of variables capturing various colonial activities: distance to colonial capitals, distance to the nearest colonial city, railway access, cash crop cultivation, mining, and Catholic mission presence. Our results are robust to excluding colonial variables that might be endogenous to missionary activity (see Supplementary Material, Table A9).

##### Individual Variables

In line with earlier studies (e.g., Nunn 2014; Cagé and Rueda 2016), we control two individual variables that are of high importance for dependent variables and that are not affected by post-treatment bias. They are gender as a binary variable indicating whether the respondent is *female* or not, and the *age* of the respondent as well as its squared value. These individual controls help reduce measurement uncertainty. However, our results do not depend on the inclusion of these control variables (see Supplementary Material Tables A10, A8).

## Results

### Education

We examine whether shared national identity leads to higher levels of human capital, which we proxy with the educational attainment variables. We first estimate regression models with two different education variables, a binary variable, primary school completion, for Models 1–3, and a more fine-grained continuous variable, years of education, for Models 4–6. Moreover, we include our three measures of British and non-British mission settlement in the analysis. Models 1 and 4 employ the binary mission dummies, while Models 2 and 5 use the number of missions in a locality, and models 3 and 6 the time since the establishment of the first mission.

All six models in Table 1 display a positive and statistically significant effect of British missions.^13^ These results are also substantively significant. Consider Model 1. The coefficient on the British mission dummy implies that the presence of a mission leads to a 3.8 percentage point increase in the probability of completing a primary education. As a further example, consider Model 6. An extra 100 years of British missionary presence leads to an increase of 1.1 years of education. Since the average number of years of schooling is about 8, the extra year represents a significant increase in education. Finally, an important result is that the coefficient on non-British missions is not significant, suggesting that non-British missions did not increase educational attainment more than areas without missions.^14^

**Table 1:** Long-term effects of protestant missions on education outcomes in British Africa

	Primary education			Years of education		
	(1)	(2)	(3)	(4)	(5)	(6)
British Mission [Dummy]	$$0.039^{***}$$			$$0.530^{***}$$		
	(0.008)			(0.086)		
Non-British Mission [Dummy]	0.000			0.054		
	(0.008)			(0.091)		
British Mission [Count]		$$0.015^{***}$$			$$0.200^{***}$$	
		(0.003)			(0.031)	
Non-British Mission [Count]		$-0.003$			$-0.037$	
		(0.002)			(0.022)	
British Mission [Time]			$$0.096^{***}$$			$$1.117^{***}$$
			(0.018)			(0.207)
Non-British Mission [Time]			$-0.013$			$-0.016$
			(0.017)			(0.193)
Sample restriction	100 km	25 km	25 km	100 km	25 km	25 km
Individual controls	$\checkmark$	$\checkmark$	$\checkmark$	$\checkmark$	$\checkmark$	$\checkmark$
Colonial controls	$\checkmark$	$\checkmark$	$\checkmark$	$\checkmark$	$\checkmark$	$\checkmark$
Precolonial controls	$\checkmark$	$\checkmark$	$\checkmark$	$\checkmark$	$\checkmark$	$\checkmark$
$R^2$	0.219	0.231	0.229	0.251	0.249	0.246
Adj. $R^2$	0.219	0.230	0.228	0.250	0.248	0.244
Num. obs	48, 250	28, 894	28, 894	48, 250	28, 894	28, 894

*Note:* OLS with colony and wave fixed-effects, and robust standard errors clustered by sampling location. Sample restricted to indicated distance from Protestant missions. Mission variables refer to Protestant stations within 25 km radius from respondent and capture their presence, their number, respectively, the time since the first establishment. Precolonial controls include altitude, terrain ruggedness, agricultural suitability, population density, malaria burden, access to waterways, centralization, high gods, polygamy, slave trade, and distances from the coast as well as Muslim centers; colonial controls include distances from the colonial capital, cities, railway access, and Catholic mission presence; individual controls include gender, age, and age squared (details in text). *0.05; **0.01; ***0.001

**Table 2:** Long-term effects of protestant missions on democratic attitudes in British Africa

	Support for democracy			Support for elections		
	(1)	(2)	(3)	(4)	(5)	(6)
British Mission [Dummy]	$$0.035^{***}$$			$$0.067^{**}$$		
	(0.008)			(0.021)		
Non-British Mission [Dummy]	$$-0.022^{**}$$			$$-0.044^{*}$$		
	(0.008)			(0.020)		
British Mission [Count]		$$0.011^{***}$$			$$0.023^{**}$$	
		(0.003)			(0.008)	
Non-British Mission [Count]		$$-0.007^{***}$$			$$-0.009$$	
		(0.002)			(0.006)	
British Mission [Time]			$$0.095^{***}$$			$$0.183^{***}$$
			(0.016)			(0.043)
Non-British Mission [Time]			$$-0.067^{***}$$			$$-0.085$$
			(0.016)			(0.045)
Sample restriction	100 km	25 km	25 km	100 km	25 km	25 km
Individual controls	$\checkmark$	$\checkmark$	$\checkmark$	$\checkmark$	$\checkmark$	$\checkmark$
Colonial controls	$\checkmark$	$\checkmark$	$\checkmark$	$\checkmark$	$\checkmark$	$\checkmark$
Precolonial controls	$\checkmark$	$\checkmark$	$\checkmark$	$\checkmark$	$\checkmark$	$\checkmark$
$R^2$	0.055	0.048	0.049	0.030	0.031	0.032
Adj. $R^2$	0.055	0.047	0.048	0.029	0.030	0.030
Num. obs	48, 250	28, 894	28, 894	48, 250	28, 894	28, 894

*Note:* OLS with colony and wave fixed-effects, and robust standard errors clustered by sampling location. Sample restricted to indicated distance from Protestant missions. Mission variables refer to Protestant stations within 25 km radius from respondent and capture their presence, their number, respectively, the time since the first establishment. See Table 1 for a list of control variables. *0.05, **0.01, ***0.001

We furthermore consider whether the results are driven by outlying cases. Therefore, we implement leave-one-out regressions, by dropping all respondents from one colony at a time and re-estimating the above models. Our findings are stable across the re-estimated models. The test results are summarized in the Supplementary Information (Figures A1-A2).

Another concern is the radii we use the delimit our sample and to define the mission sphere of influence. While we follow common practice, we show that our results hold when a wider sphere of influence is considered as well as when we do not impose geographic restrictions on the sample. The results are shown in the Supplementary Material (Tables A11–A13).

We also show that our results are robust to a variety of meaningful adjustments of the included control variables. The results hold when we omit individual control variables (see Supplementary Material Tables A8). They also do not change when we include more precise controls about the colonial economy, which are only available for a subset of our cases (see Supplementary Material, Table A10). The results thus support H1: shared British national identity between state and mission leads to higher educational attainment.

### Support for Democracy

The second hypothesis states that respondents in areas that had British colonial missions have higher levels of support for democracy than respondents who live in areas that had non-British missions. We begin by estimating two models in which support for democracy is the dependent variable (see Table 2). Model 1 uses the binary mission indicators to estimate linear probability models of how the historical exposure to Protestant missions affects contemporary support. In line with our hypothesis, the model reveals a positive and statistically significant effect of exposure to a British mission. The effect of non-British missions is smaller, but also statistically significant. Interestingly, it points in the opposite direction. Models 2 and 3 use our two alternative continuous measures of exposure. We find the same pattern and support for our hypothesis as in the first model. This result shows the mission coming from the same nation as the colonial power is a contributing factor for long-run support for democracy. This in turn may suggest that national origins are an enabling condition for the efficacy of missions as political agents.

Substantively, the effect sizes in both models are considerable. As the dependent variable is binary, the effects can be interpreted as percentage point changes. According to Model 1, the presence of a British mission increases the probability that a respondent supports democracy by 3.0 percentage points. Model 3 shows that the probability increases over time, 8.6 percentage points for every 100 years, or about one percentage point every 12 years.

In Models 4–6, we estimate the effect on support for elections, our alternative outcome to assess pro-democratic attitudes. The variable is measured on a more fine-grained five-point scale. As expected, we find virtually the same effects, and they are almost twice as large in magnitude. However, the unexpected results that non-British missions even reduce democratic support disappears. While coefficients still point in a negative direction, they are statistically insignificant. This suggests that non-British missions are likely to have some harmful effects for support for democracy, but they are not as large or consistent as the pro-democratic effects of British missions.

The results hold in a wide range of sensitivity and robustness checks. We confirm logit specification leads to similar results (see Supplementary Material, Table A2). The results also do not depend on widening the mission perimeter or on the sample restriction we imposed to reduce the risk of confounders (see Tables A11–A13). They are also robust to removing individual control variables from the models (see Table A8) as well as adding additional controls on the colonial economy, which are available only for a subset of the data (see Table A10). Furthermore, leave-one-out regressions show removing respondents from single colonies does not affect the results (see Figures A3–A4). Finally, estimating separate models leads to virtually the same results for British missions (see Table A14) while the effect of non-British mission becomes insignificant (see Table A15). Overall, H2 is also supported: shared national identity between the British colonial state and British Protestant missions leads to long-run higher levels of support for democracy.

### Robustness of Main Results

**Religious Competition** Protestant and Catholic missions in Africa competed over converts in colonies. Studies have shown how the colonial context shaped the dynamics of such competition: Protestants induced higher long-term effects on education where they competed with Catholics that were shielded by colonial governments (i.e., most non-British colonies) (Gallego and Woodberry 2010). In this section, we extend this line of argumentation and test whether the presence of Catholic missions shaped the legacies of Protestant missions in British colonies (see also Lankina and Getachew 2013).

To test whether Catholic missions exerted competitive pressures on Protestant missions in British colonies, and thereby altered their educational and democratic legacies, we re-estimate our main models after splitting our sample into areas with and without a Catholic mission present. The full results are included in the Supplementary Material (see Tables A16 & A17). They show that the British missions increased education levels and support for democracy in the long run whether Catholic missions were present or not. However, in the presence of Catholic missions, the effects of British Protestant missions are about twice as large as in areas without Catholic missions. These findings suggest that competitive pressures induced British Protestant missions to more strongly lean on their close ties to British colonial governments or that Catholic missions adopted practices from Protestant missions. As such, competition either moderates our proposed mechanism or unfolds independently.

**Policy Change** Another factor that moderates our main effect would be changing colonial policy over time. Colonialism in Africa lasted hundreds of years; colonial policy obviously changed as a result of changes in the international system, as well as the domestic politics in both colonized and colonizer countries. While the paper does not focus on these changes over time, we acknowledge the importance of policy choices and provide a basic examination of how important policy shifts can affect our main result. An important policy change with respect to education policy was the result of Phelps Stokes commission tours to Africa underscoring the importance of formal education for the colonial project (Becker and Schmitt 2023; Steiner-Khamsi and Quist 2000). The remainder of the decade saw a series of memorandums further supporting the importance of education to colonial policy (Becker and Schmitt 2023).

To account for the effects of colonial policy on our main results, we split the sample between missions established before and after 1920 in Supplementary Material Table A18. We find that British Protestant missions established before 1920 led to an increase in both long-run education and support for democracy, consistent with the main results. Non-British Protestant missions do not show significant impacts. Perhaps surprisingly, British Protestant missions established after 1920 do not lead to significant impacts in education and support for democracy, despite the significantly greater resources the British colonial state put into education. It is difficult to determine whether these results are due to policy changes or simply because missions that were established earlier had more time to establish capacities for educational development. At the very least, these results may suggest that mission longevity exerts a strong effect on long-run education and support for democracy, beyond the effect of state resources.^15^

**Democratization** While classic modernization theory posits that the formation of a middle class spurs democratization, more recent literature has argued that increased education does not lead to greater democracy in autocracies, for example, because a more educated, richer middle class is beholden to the state for jobs and other forms of patronage (Chen 2013; Rosenfeld 2020). Regime type thus serves as a potential moderator between education and support for democracy.

We examine the role of regime type in Supplementary Material Tables A19 and A20 by splitting our sample based on countries’ democratic performance in 1995, and thus at the end of the third wave of democratic transitions.^16^ We focus on democratic performance in 1995 to distinguish countries that are successfully democratizing from those that remain more autocratic. We take into account arguments about the non-democratic roots and system-maintaining function of education (Paglayan 2021). We find that our results hold regardless of regime type. The presence of a British Protestant mission leads to increased education and support for democracy in both low- and high-democracy contexts, although the statistical evidence is slightly stronger for the prior set of countries.

### Mediation Analysis

To provide further evidence of mission support for democracy effects through education, we conduct a mediation analysis. We begin by testing the underlying proposition that more educated individuals are also more supportive of democracy. In line with the existing literature (Evans and Rose 2012), Table 3 provides strong support for this. Both primary school completion and years of education exert a substantive effect on support for democracy as well as support for elections. Primary school completion increases the probability of support for democracy by almost 14 percentage points and support for elections by over 0.1 points. Similarly, every additional year of education is associated with a 1.6 percentage point increase in the probability to support democracy and 0.014 points to support elections. To determine the degree to which the political legacy of missions results from the pro-democratic effects of their education offers, we estimate a range of mediation models.

In the mediation models, we use the binary variable that indicates the presence of British missions as the exogenous variable, years of education as mediator, and support for democracy, and alternatively elections, as the dependent variable. We estimate the models with and without control variables.

**Table 3:** Baseline results of education mechanism

	Support for democracy		Support for elections	
	(1)	(2)	(3)	(4)
Primary education	$$0.141^{***}$$		$$0.103^{***}$$	
	(0.006)		(0.016)	
Years of education		$$0.017^{***}$$		$$0.015^{***}$$
		(0.001)		(0.002)
Sample restriction	100 km	100 km	100 km	100 km
Individual controls	$\checkmark$	$\checkmark$	$\checkmark$	$\checkmark$
Colonial controls	$\checkmark$	$\checkmark$	$\checkmark$	$\checkmark$
Precolonial controls	$\checkmark$	$\checkmark$	$\checkmark$	$\checkmark$
$R^2$	0.069	0.076	0.030	0.031
Adj. $R^2$	0.068	0.075	0.029	0.030
Num. obs	48, 250	48, 250	48, 250	48, 250

*Note:* OLS with colony and wave fixed-effects, and robust standard errors clustered by sampling location. Sample restricted to indicated distance from Protestant missions. See Table 1 for a list of control variables. *0.05, **0.01, ***0.001

The results of the mediation analysis attest to a statistically significant average causal mediation effect on support for democracy (see Supplementary Material, Figure A5, top panel). This indirect effect, of missions on support for democracy through education, accounts for between one and two-thirds of the total effect, of missions on support for democracy, depending on whether control variables are included.

This is a very high proportion given that the mediation analysis constitutes a conservative test of the education mechanism. It only picks up whether support for democracy is mediated by the education of the individual respondent. However, mission education is known to have important spill-over effects (Wantchekon et al. 2015). Our mediation analysis is conservative as it does not capture how the education of parents and grandparents might affect younger family members independently of the education of those members. It also does not capture spill-over effects into the wider community. As such, the mediation analysis provides strong support for a large and substantive effect of the education mechanism.

However, it should be noted that mediation results for our alternative dependent variable, support for elections, are less strong but remain sizeable (see Supplementary Material Table Figure A5, bottom panel). A mediation model without controls shows that years of education mediate 28.4% of the total effect of British missions on support for elections. Once controls are included, this proportion is reduced to 10.0%.

### Addressing Endogeneity of Mission Settlement

In this section, we address endogeneity concerns that result from selection biases of missions entering territories after they have been colonized. Colonial states regulated whether missionaries could enter their territories and where they set up stations (see Becker 2022). This might induce selection biases that confound differences between British and non-British missions because British missions were less likely to be deterred from entering British colonies and more likely to be given favorable mission settlement areas relative to non-British missions. While the above analyses control for a variety of factors that may affect endogenous mission settlement, it is possible that some endogeneity remains. To address these outstanding concerns, we exploit the fact that many mission stations were installed before British colonization. Among these early missions, the above endogeneity concerns do not apply.

**Table 4:** Long-term effects of early protestant missions on contemporary outcomes

	Primary education	Years of education	Support for democracy	Support for elections
	(1)	(2)	(3)	(4)
British Mission [Dummy]	$$0.060^{***}$$	$$0.674^{***}$$	$$0.030^{*}$$	$$0.081^{*}$$
	(0.018)	(0.187)	(0.012)	(0.037)
Non-British Mission [Dummy]	0.016	$0.254^{*}$	$-0.017$	$-0.015$
	(0.010)	(0.120)	(0.011)	(0.028)
Sample restriction	100 km	100 km	100 km	100 km
Individual controls	$\checkmark$	$\checkmark$	$\checkmark$	$\checkmark$
Colonial controls	$\checkmark$	$\checkmark$	$\checkmark$	$\checkmark$
Precolonial controls	$\checkmark$	$\checkmark$	$\checkmark$	$\checkmark$
$R^2$	0.226	0.246	0.069	0.027
Adj. $R^2$	0.225	0.245	0.068	0.026
Num. obs	29, 798	29, 798	29, 798	29, 798

*Note:* OLS with colony and wave fixed-effects, and robust standard errors clustered by sampling location. Sample restricted to indicated distance from early Protestant missions, i.e., established before British colonization. Mission variables indicate the presence of early Protestant stations within 25 km radius from respondent. See Table 1 for a list of control variables; in addition, the presence of an early Catholic mission is controlled for (see text for details). *0.05, **0.01, ***0.001

For this purpose, we restrict our sample to locations where missions were set up before British colonization and compare them to proximate locations without any missions.^17^ The results corroborate our main findings (see Table 4). Early British missions have sizeable effects on contemporary education and pro-democratic attitudes. At the same time, we find no evidence that early non-British missions have any long-term effects.

## Examining Scope Conditions

We now turn to examining the scope conditions of our argument. In particular, we analyze whether our theory of national origins is specific to Protestant missions in British Africa, or some combination of alternative Christian denominations and colonizer countries also follow the same national origins logic. We focus specifically on Catholicism and on the French. As the major alternative Christian denomination respectively colonial power, both are intuitive choices. Like Protestant missions, Catholic missions were also tasked with providing education and schooling, and it is thus potentially true that through schooling, the burgeoning of democratic attitudes also sprung forth. Likewise, the French differed from the British across a panoply of legal, cultural, and social institutions. As such, it is plausible that some combination of these differing institutions may have also led to increased education and long-run democratic attitudes when paired with Protestant (or perhaps Catholic) missions.

We identify Catholic mission locations (see Fig. A6), and in line with our previous analysis, contemporary education outcomes and democratic attitudes and is less informative. We acknowledge that coding the nationality of Catholic missions according to the seat of their sending society is due to the centralized organization of the Catholic Church less informative than in the case of Protestant missions. This warrants a more cautious weighing of the corresponding evidence. Overall, 13 French colonies are part of our sample.^18^

### British Missions in French Africa

We first examine whether British missions, which were so effective at spurring both education and democratic attitudes in British Africa, were also successful at spreading both outcomes in French Africa. If we find that British missions improved both education and democratic outcomes in French Africa, such a result would suggest that it is British missions per se, and not the national linkages among compatriots, that are driving the result. Furthermore, such a result may also call into question whether this is a British story. Perhaps national origins matter regardless of the identity of the colonizing country.

**Table 5:** Long-term effects of british missions in french colonies

	Primary education	Years of education	Support for democracy	Support for elections
	(1)	(2)	(3)	(4)
British Mission [Dummy]	$-0.012$	$-0.086$	$-0.038^{*}$	$-0.010$
	(0.021)	(0.245)	(0.018)	(0.042)
Non-British Mission [Dummy]	0.005	0.214	$$-0.055^{***}$$	$$-0.043$$
	(0.018)	(0.220)	(0.015)	(0.036)
Sample restriction	100 km	100 km	100 km	100 km
Individual controls	$\checkmark$	$\checkmark$	$\checkmark$	$\checkmark$
Colonial controls	$\checkmark$	$\checkmark$	$\checkmark$	$\checkmark$
Precolonial controls	$\checkmark$	$\checkmark$	$\checkmark$	$\checkmark$
$R^2$	0.301	0.318	0.055	0.044
Adj. $R^2$	0.300	0.316	0.053	0.041
Num. obs	12, 733	12, 733	12, 733	12, 733

*Note:* OLS with colony and wave fixed-effects, and robust standard errors clustered by sampling location. Sample restricted to indicated distance from Protestant missions. Mission variables indicate the presence of a Protestant station within 25 km radius from respondent. See Table 1 for a list of control variables. *0.05, **0.01, ***0.001

To test these alternative scope conditions, we consider the effects of British and non-British Protestant missions in French Africa. We identify mission settlement (see Fig. 1), and our education and democratic attitude outcomes, similarly to our previous analysis. We find that neither British missions nor non-British missions have any long-term effect on either primary schooling or years of education (see Table 5). Moreover, British missions are negatively and significantly correlated with support for democracy. That is, in French Africa, respondents who lived in areas which had British missions are even less likely to support democracy than in areas that had no mission settlement. Finally, the relationship between British missions and support for elections is negative yet not significant.

Overall, these results seem to suggest that national origins do not necessarily translate to French colonies, nor is it British missions per se that are responsible for long-run democratic attitudes.

### Catholic Missions in British Africa

We next consider Catholic missions in British Africa. On the one hand, one might expect Catholics to also benefit from national linkages and thus to equally spur education and support for democracy as Protestant missions. This would suggest that our argument is not a uniquely British Protestant story. On the other, national linkages might have been inconsequential as Catholicism was not the major religion in Britain and the Catholic Church was more centrally organized, supplying missionaries with more independent support. Null effects would corroborate the scope conditions, on which we based our argument.

To test this potential scope condition, we run a similar analysis with respect to Catholic missions (both British and non-British) in British Africa. Our main data source is the Atlas Hierarchichus (Streit 1929), which we digitized specifically for this purpose.^19^ We identify mission settlement (see Figure A6), and our education and democratic attitude outcomes, similarly to our previous analysis. We find that British Catholic missions neither increased primary education or years of schooling, while non-British missions have a positive long-term impact (see Table 6). Moreover, British Catholic missions are negatively and significantly related to both measures of democratic attitudes, and no effect can be discerned for non-British Catholic missions. These results suggest that national origins of Catholic missions in British Africa did not lead to the promotion of both education and long-run democratic attitudes as it did for Protestant missions. In line with our theory, it is the combination of national origins and Protestantism that matters.

**Table 6:** Long-term effects of catholic missions in British colonies

	Primary education	Years of education	Support for democracy	Support for elections
	(1)	(2)	(3)	(4)
British Mission [Dummy]	$$-0.042^{*}$$	$$-0.349$$	$$-0.063^{**}$$	$$-0.182^{**}$$
	(0.021)	(0.212)	(0.020)	(0.060)
Non-British Mission [Dummy]	$$0.078^{***}$$	$$0.991^{***}$$	0.014	0.013
	(0.008)	(0.093)	(0.008)	(0.021)
Sample restriction	100 km	100 km	100 km	100 km
Individual controls	$\checkmark$	$\checkmark$	$\checkmark$	$\checkmark$
Colonial controls	$\checkmark$	$\checkmark$	$\checkmark$	$\checkmark$
Precolonial controls	$\checkmark$	$\checkmark$	$\checkmark$	$\checkmark$
$R^2$	0.220	0.258	0.056	0.032
Adj. $R^2$	0.220	0.257	0.055	0.031
Num. obs	42, 846	42, 846	42, 846	42, 846

*Note:* OLS with colony and wave fixed-effects, and robust standard errors clustered by sampling location. Sample restricted to indicated distance from Catholic missions. Mission variables indicate the presence of a Catholic station within 25 km radius from respondent. See Table 1 for a list of control variables. *0.05, **0.01, ***0.001

It is important to note that the number of British Catholic missions was small. In our data source, there is in fact only one British Catholic mission society, Saint Joseph’s Missionary Society of Mill Hill, and it ran 22 mission stations in the Belgian Congo, British Cameroon, Kenya, and Uganda. As such, our findings might be driven by idiosyncrasies of the society. Furthermore, our data does not account for the exact staff composition at mission stations and therefore ignores that in some cases the homeland of missionaries did not correspond to the origin of the mission society, such as the presence of Irish missionaries at some French Catholic mission stations in Southern Nigeria who was generally perceived as closely allied with the state. Taking this nuance into account might be particularly relevant to fully understand the exact impact of the small number of British Catholic missionaries and cautions against drawing any definite conclusions about them from the results presented here. That being said, given the available data, the findings presented here are in line with our overarching argument.

### Catholic Missions in French Africa

We next examine Catholic missions in French Africa. Relations between the French state and colonial missions were markedly different from that of the British church and state. The French state was avowedly secular and was at times outwardly hostile to Catholic missionaries (Conklin 2000) after 1870 (Kuru 2007). Instead, the French state favored the creation of secular public schools, which they used to cultivate a small class of African elites to serve the lower levels of colonial administration (Conklin 2000). This secular model should have distinct implications for our argument. On the one hand, we may find that the presence of Catholic missions in French Africa leads to an increase in long-run educational attainment, since missions did provide education. On the other hand, we do not expect to see mission presence to lead to long-run support for democracy, since the separation of church and state in French Africa implies the decoupling of missionary education with democratic and civic values. This analysis can therefore speak to how alternative modes of church-state relations affect the link between missionary activity, education, and support for democracy.

To test this potential scope condition, we run a similar analysis with respect to Catholic missions (both French and non-French) in French Africa. The results suggest that the mechanisms that work for British Protestants in British Africa do not work for French Catholics in French Africa (see Table 7). While French missions did lead to an increase in education, we also find a similar increase for non-French missions. Moreover, the French mission settlement did not lead to a long-run increase in democratic attitudes. This result, as well as the previous results, suggests that the case of Protestant missions in British Africa is the appropriate scope condition for national origins to affect both education and democratic attitudes.

Broadly, our results speak to literature on how various models of church-state relations (Stepan 2000; Bulutgil 2022) affect the link between missionary activity, education, and democratization. In particular, our results suggest that when church and state are more integrated (as in British Protestant missions in British Africa), educational attainment via missions leads to long-run support for democracy, plausibly because democratic and civic values from the state percolate into missionary education. Whereas in places adhering to a secular model, missionary education does not lead to long-run support for democracy because education is not necessarily imbued with the civic and democratic ideals of the state.

**Table 7:** Long-term effects of catholic missions in French colonies

	Primary education	Years of education	Support for democracy	Support for elections
	(1)	(2)	(3)	(4)
French Mission [Dummy]	$$0.077^{***}$$	$$1.008^{***}$$	0.025	0.036
	(0.018)	(0.200)	(0.015)	(0.041)
Non-French Mission [Dummy]	$$0.129^{***}$$	$$1.304^{***}$$	$$-0.018$$	$$-0.043$$
	(0.020)	(0.231)	(0.016)	(0.039)
Sample restriction	100 km	100 km	100 km	100 km
Individual controls	$\checkmark$	$\checkmark$	$\checkmark$	$\checkmark$
Colonial controls	$\checkmark$	$\checkmark$	$\checkmark$	$\checkmark$
Precolonial controls	$\checkmark$	$\checkmark$	$\checkmark$	$\checkmark$
$R^2$	0.294	0.321	0.070	0.043
Adj. $R^2$	0.292	0.319	0.068	0.041
Num. obs	15, 225	15, 225	15, 225	15, 225

*Note:* OLS with colony and wave fixed-effects, and robust standard errors clustered by sampling location. Sample restricted to indicated distance from Catholic missions. Mission variables indicate the presence of a Catholic station within 25 km radius from respondent. See Table 1 for a list of control variables. *0.05, **0.01, ***0.001

## Conclusion

A large and growing literature has examined the positive impacts of British colonialism (Lee and Paine 2019; Acemoglu et al. 2001; Frankema 2012) and Protestant missions (Gallego and Woodberry 2010; Lankina and Getachew 2012; Wantchekon et al. 2015) on long-run developmental outcomes. As a result, Protestantism was a significant catalyst underpinning the positive effects of British colonialism. We posited that this simple dichotomy masks important heterogeneities in how the British colonial state engaged with colonial missions. Our argument emphasized the importance of shared national origins between the British state and colonial mission as a means to induce cooperation from both sides, owing to their shared interest in improving educational attainment. British Protestant missions received greater support from the British colonial state than non-British missions, and as a result, they were able to increase educational attainment and, though unintended, support for democracy amongst the local population in ways that persist into the present. Quantitative evidence from 19 former British colonies in Africa supports the argument. Our work further explicated church-state relations in order to provide a deeper understanding of how this dynamic influenced long-run political and economic development.

Future research can further tease out the complex dynamics between mission and state and tie these relations to a variety of outcomes, including state capacity, female empowerment, and grassroots anticolonial movements, to name a few. Two potential avenues stand out. First, the missions were increasingly Africanized, with natives taking over the spread, organization, and local religious hierarchies of the church. Indeed, Africa today is home to a vibrant religious community and to religious organizations. The impacts of Africanization on the outcomes mentioned above may be a fruitful area of research. A second avenue of research may examine how missionary activity led to fledgling independence movements across the postcolonial world that eventually shepherded their countries into independence. An irony of colonialism is that many of the native elites who opposed it were the product of a privileged colonial education. Further teasing out the link between mission education and independence movements through quantitative analysis may be especially fruitful.

Another area for future research concerns the content of education, in particular the transmission of civic and democratic values. It stands to reason that close Church-state relationship unleashed “British values” as British missionaries had a greater capacity to imbue their values on local populations in British colonies where they received more governmental support. At the same time, educational content might also be shaped by ties to colonial governments, who increasingly sought to influence mission education in the twentieth century, but also due to variation in the amicability and adversity in the relations between different mission societies and colonial governments. Relatedly, the legacies of government schools, though less prevalent than mission schools in British colonies, have only received scant attention despite their importance for civic and elite education. Unpacking the content of education, and differences between mission and government schools, would be valuable extensions of the current scholarship.

Overall, our findings suggest that British Protestant missions had a decisive impact on the long-run economic and political development of British colonies. They are therefore key to understanding why former British colonies are often richer and more democratic than countries colonized by other powers. More generally, our findings urge for a closer investigation of national networks in colonial empires, including collaborations with other actors, e.g., merchants. Such efforts hold great promise to improve our understanding of contemporary African and international politics.

## Supplementary Information

Below is the link to the electronic supplementary material.

Supplementary file 1 (pdf 482 KB)

## Data Availability

Data on 770 Catholic mission stations from the newly georeferenced Atlas Hierarchicus (Streit, 1929) is available through the Harvard Dataverse (https://doi.org/10.7910/DVN/JAVD83)

## Footnotes

1 It is important to note that Africans constituted the majority of mission staff, ran many outstations, and decisively advanced education and Christianity (Jedwab et al. 2022). Nevertheless, we focus on mission stations run by Western missionaries, who functioned as bottlenecks when it came to collaboration with the state.

2 Additional analyses of survey respondents in 13 French colonies explore the scope conditions of our argument.

3 It is important to note that the link between Protestant missions and democracy is still a topic of robust debate. In a replication of Woodberry’s study, Nikolova and Polansky (2021) use more extensive data to show that the original results do not hold. Our findings suggest that the replication failure might be due to differences among British and Non-British Protestants, which neither of the two studies consider.

4 In the general sense, the link between missionary presence and educational attainment is documented in the literature. Indeed, Dahlum and Wig (2019) use the presence of Christian missions in Africa as an instrumental variable for educational attainment.

5 It is important to note that Paglayan (2021) finds most of the expansion of primary education took place before democratization. This further supports our claim that missions expanded education in the non-democratic colonial context, thus laying the groundwork for the spreading of beliefs that paved the way for democratization.

6 There is also some evidence that the particulars of the British curriculum, in particular a focus on humanistic education, may have further established pro-democratic sentiment in the colonies (Martin 2023).

7 Our analysis of British Africa covers Bechuanaland, British Cameroon, Egypt, Swaziland, Gold Coast, British Togoland, Sierra Leone, Kenya, Uganda, Basutoland, South Africa, Nyasaland, South West Africa, Nigeria, Sudan, Tanganyika, Zanzibar, Northern Rhodesia, and Southern Rhodesia.

8 We present linear model results in the main text and logit results of the main models in the Supplementary Material (see Table A2).

9 The results are robust even when the sample is further restricted to 50 km or not restricted at all (see Supplementary Material, Tables A11 and A12).

10 The 25 km radius considers typical walking distances and the distribution of outstations (Nunn 2014). These outstations are not included in the WMA, which focuses on main stations that usually had a European resident. However, outstations were set up in close proximity of main stations. Furthermore, our data does not allow us to account for the exact staff composition of mission stations. While mission stations were occasionally home to missionaries from multiple nations, we are not aware of any source that would provide such fine-grained information for all of Africa, and the origin of the mission society is arguably most relevant to understand linkages with colonial governments.

11 In our main analysis Irish and Scottish missions are coded as British; our results are robust when they are coded as non-British missions (see Supplementary Material, Table A7).

12 We choose 1930 as the reference year as the Catholic data we use for control variables and additional analyses is from the year 1929.

13 The results of models 1–3 in Table 1 hold when logit specifications are used (see Supplementary Material, Table A2).

14 Separately estimated models lead to virtually the same results for British missions (see Table A14) while the effect of non-British mission becomes significant but remains much smaller than the effect of British missions (see Table A15).

15 Another possible explanation is the occurrence of the two world wars, which weakened European colonial powers and led to the emergence of anticolonial movements, with potential implications for education policy and practices.

16 We use V-Dem’s polyarchy score (Coppedge et al. 2020) and separate the most democratic countries in our sample (top half) from the remaining less democratic countries.

17 As before, we use dummy variables to indicate the presence of a mission within 25 km of respondents’ locations, and proximate locations are any locations within 100 km of early mission stations. Locations where missions were set up after British colonization are omitted.

18 The included French African colonies are Algeria, Dahomey, French Togoland, Upper Volta, French Cameroon, Ivory Coast, Gabon, Guinea, French Sudan, Mauritania, Niger, Senegal, and Tunisia.

19 Georeferenced data on the 770 Catholic mission stations included in the Atlas Hierarchicus is available through the Harvard Dataverse (https://doi.org/10.7910/DVN/JAVD83).
