# Supplementary material for: Between God and Nation: The Colonial Origins of Democracy Support in British Africa
Source: Stud Comp Int Dev. 2024 Dec 6;61(1):136–67. doi: 10.1007/s12116-024-09450-2 (PMC13090240; doi:10.1007/s12116-024-09450-2)
Supplement: Supplementary file 1 — (pdf 482 KB) [file 12116_2024_9450_MOESM1_ESM.pdf]

APPENDIX

A Data

Table A1 Descriptive Statistics of Analytical Dataset

Statistic	N	Mean	St. Dev.	Min	Pctl(25)	Pctl(75)	Max
Primary Education	48,250	0.723	0.448	0	0	1	1
Years of Education	48,250	8.142	4.582	0	3	12	19
Support for Democracy	48,250	0.692	0.461	0	0	1	1
Support for Elections	48,250	4.126	1.302	1	4	5	5
Female	48,250	0.499	0.500	0	0	1	1
Age	48,250	37.035	14.810	18	26	45	105
British Mission [Dummy]	48,250	0.351	0.477	0	0	1	1
Non-British Mission [Dummy]	48,250	0.369	0.483	0	0	1	1
Catholic Mission [Dummy]	48,250	0.339	0.473	0	0	1	1
British Mission [Count]	48,250	0.938	1.763	0	0	1	14
Non-British Mission [Count]	48,250	0.969	2.220	0	0	1	18
Catholic Mission [Count]	48,250	0.518	0.875	0	0	1	5
British Mission [Time]	48,250	0.112	0.239	0	0	0.1	1
Non-British Mission [Time]	48,250	0.157	0.283	0	0	0.2	1
Catholic Mission [Time]	48,250	0.115	0.221	0	0	0.2	1
Coast (Dist.)	48,250	421.877	318.969	0.005	142.912	657.690	1,238.010
Water	48,250	0.402	0.490	0	0	1	1
Ruggedness	48,250	89.099	136.776	0.000	14.160	99.815	984.216
Altitude	48,250	901.149	621.555	−38	276	1,327	3,939
Malaria Burden	48,250	2.381	1.122	0	2	3	4
Agriculture Suitability	48,250	3,389.356	2,148.599	0	2,045	4,709.2	9,929
Population Density	48,250	35.523	84.208	0	2.4	28.0	906
Centralization	48,250	0.635	0.482	0	0	1	1
Polygamy	48,250	0.590	0.492	0	0	1	1
High Gods	48,250	0.085	0.278	0	0	0	1
Muslim Centers (Dist.)	48,250	752.778	477.477	0.135	436.447	995.577	2,399.628
Transatlantic Slavery	48,250	0.086	0.266	0	0	0.03	7
Colonial capital (Dist.)	48,250	232.499	218.640	0.000	69.605	336.408	1,414.950
Colonial City (Dist.)	48,250	380.316	320.176	0.090	122.101	579.577	1,379.306
Railway Access	48,250	0.240	0.427	0	0	0	1
Cash Crop	33,619	0.420	0.494	0.000	0.000	1.000	1.000
Mining	33,619	0.072	0.258	0.000	0.000	0.000	1.000

Precolonial variables The most basic pre-colonial variables we account for relate to the geography of the locations Protestant missions settled in. Generally, locations that were easily accessible and had a hospitable environment were preferred by missions for settlement. Almost all missionaries arrived in Africa by sea. Venturing inland was only possible at considerable cost. Missionaries

therefore preferred to settle near the coast, both because it was less costly to do so, but also because staying near the coast provided missionaries with more opportunities for economic activity, such as trade. To account for this, we control for the distance to the nearest point on the *coast*.[21]

The difficulty of inland endeavors depended on a variety of factors. Africa has few large navigable waterways, but locations close to them were much easier to access. We code locations within 25 kilometer as having access to a navigable waterway (*water*). Similarly, locations at higher levels of *altitude*, while often more hospitable due to a lower disease burden, were usually harder to reach. We therefore control for each location's elevation above sea-level. Another factor that could make access more difficult was terrain *ruggedness*, data on which we derive from Shaver, Carter, and Shawa (2016). Their measure is based on a grid-cell approach (1km^2) that indicates the degree to which the elevation of each cell differs from that of its neighbors. Ruggedness affects where missionaries settled because it was harder to access and set up stations in more rugged terrain (Nunn & Puga, 2012). Furthermore, rugged terrain is detrimental for agriculture and can sustain only smaller populations. Lower numbers of prospective converts decreased the incentive of missionaries to control the area.

Missionaries often preferred elevated territories as they were associated with lower exposure to malaria-transmitting mosquito. We directly account for the *malaria burden* drawing on a historical map frequently used in epidemiological studies. The map by Lysenko and Semashko (1968) indicates the endemicity of malaria in 1900, ranging from malaria-free (0) to holoendemic (5). The relationship between mission settlement and malaria incidence is obvious: missions preferred to settle in areas with lower malaria incidence.

Finally, missionaries usually relied on farming for their own sustenance. *Agriculture suitability* was therefore an important determinant of where missionaries set up new stations: missions preferred to settle in places with higher levels of agricultural suitability, as they would be able to take rents from more economically profitable areas. Our measure comes from "Global Agro-ecological Zones (GAEZ v3.0)" (2011) and indicates the suitability of local conditions for the cultivation of high-level rain-fed crops.

In addition to natural factors, social geography also played an important role in the locational

choices of missionaries. As such, they preferred locations that were economically and politically more developed. This not only promised higher living standards but also more prospective converts. Following other scholars, we control for economic development relying on *population density* measures from Klein Goldewijk, Beusen, and Janssen (2010). Population density is a good proxy for urbanization and development and is increasingly used in quantitative historical work (Fresh, 2018; Stasavage, 2014). Our measure for political development is the degree of precolonial *centralization*. We construct a binary measure from Murdock (1967) that indicates whether a location was home to an ethnic group with a jurisdictional hierarchy that reached beyond the local level.

Whether missionaries could settle in a certain region and how successful they were also depended on how they were received by local populations. Religious conflict made it difficult for missionaries to advance where Islam was already dominant. The presence of Islam thus directly affects mission settlement. We account for this by including a measure of the distance to the nearest *Muslim center* in Jedwab, Meier zu Selhausen, and Moradi (2022).

Another determinant, especially of early mission stations, were Transatlantic slave networks. Missionaries often relied on the same shipping routes and were engaged in anti-slavery movements. Therefore, mission stations, especially those established during early colonization, were often set up areas affected by the Transatlantic slave trade. We operationalize the influence of the slave trade as the number of slaves per capita at the ethnic homeland level. All missions within a given ethnic homeland are coded as having the same number of slaves per capita. We use the slavery data by (Nunn, 2008) in combination with a map of ethnic homelands (Murdock, 1967) to determine the number of slaves taken. Based on population figures from (Klein Goldewijk et al., 2010), we transform these numbers into per-capita figures.

Last but not least, African demand for education played a role in where missions settled and influenced how effective they could be. As already pointed out above, one such factor was population density, as it determined the sheer number Christian missionaries could appeal to. Traditional religious beliefs and practices determined how open local populations were to Christianity. As such, the pre-existence of high gods, other "moralizing supernatural agents", could make people less receptive of the Christian god as it posed a threat to identities and cooperative arrangements (Shariff,

Norenzayan, & Henrich, 2011). Moreover, traditional religious beliefs were potential competitors to the Protestant missions, and religious competition may have spurred greater and higher-quality educational provision in these areas. We account for the presence of such high gods through a binary variable based on data from Murdock (1967). This coding takes into account the independent conversion of ethnic groups to Christianity ahead of the modern missionary movement, such as the Coptic Christians in Egypt.[22]

Furthermore, missionaries often sought to overcome norms which were considered incompatible with Christianity but were also highly valued by local populations. Becker (2021) argues that the struggle over polygamy in particular suppressed educational demand with lasting consequences for contemporary outcomes. To account for this, we use another binary variable based on Murdock (1967) to indicate whether polygamy was traditionally practiced in a given area. As there are likely other practices over which missionaries and local populations collided, we show that our results are robust to including fixed effects for ethnic homelands (see Appendix, Tables A3 and A4).

Colonial variables As mentioned above, missionaries often relied on the transportation networks of other European actors. In earlier periods, missionaries relied heavily on commercial endeavors. As European governments became more active on the continent, investments in transportation networks increased considerably and missionaries enjoyed more opportunities. This also meant that remote locations further away from the coast became more accessible.

Colonial capitals and larger cities became important transportation hubs for European activities. As such, missionaries would prefer to be closer to capital or major cities, all things equal. We therefore control for the distance to both of them. We measure the distance to each *colonial capital* based on its location in 1925. We determine the distance to the nearest *colonial city* with more than 10,000 inhabitants at the beginning of the twentieth century, based on the locations provided by Jedwab and Moradi (2016) and Becker (2022). The main infrastructure investment that colonial governments undertook was railways. Railway access made it much easier to reach inland destinations. As such, missions would prefer to settle closer to railways. We use a binary variable to capture whether locations are within 25 kilometers of a *railway*.

The location of economic activities not only influenced where missionaries could go, but also affected long-term development. Economic activities relied on local labor, and in case specific skills were required this could spur demand for education among companies and workers. Furthermore, the colonial economy also has political consequences due to its often violent nature as well as its lasting imprint on the economic structure of countries and regions.

We directly account for different economic activities during the colonial era with two binary variables that indicate whether a location produced *cash crops* or engaged in *mining* activities for export. As such, we account for two dominant economic activities whose long-term effects on economic and political development are well-established (Knutsen, Kotsadam, Olsen, & Wig, 2017; Pengl, Roessler, & Rueda, 2021; Roessler, Pengl, Marty, Titlow, & van de Walle, 2020). We use the map by Hance, Kotschar, and Peterec (1961) which indicates locations of economic activities across colonial Africa and determine whether took place within 25 kilometers of the respondent. As the map covers only parts of our sample, we employ the two variables only in additional models that are presented in the Appendix (see Tables A10 and A8).

Finally, the presence of Catholic missions fostered competition for converts and affected economic and social development in the long-run. In other words, competing religions like Catholicism forced Protestant missions to provide "consumer goods" perhaps in the form of education, in order to continue to induce potential converts to stick with them and not convert to Catholicism (Amasyalı, 2021, 2022; Lankina & Getachew, 2013). We use a several variables to control for the presence of a *Catholic mission*. They are constructed similar to the Protestant variables. First, there is a dummy that indicates the presence of a Catholic mission. Second, there is a continuous variable that captures the number of Catholic missions in the area. Third, another continuous variable indicates for how long Catholic missions have been present in the area.[23] For this purpose we digitize maps contained in the *Atlas Hierarchicus* (Streit, 1929), which provides a more complete picture of Catholic missions in interwar Africa than other historical documents.

B Further results

Table A2 Long-term Effects of Christian Missions on Contemporary Outcomes (Logit)

	Primary Education			Support for Democracy		
	(1)	(2)	(3)	(4)	(5)	(6)
British Mission [Dummy]	0.249^{***}			0.173^{***}		
	(0.049)			(0.038)		
Non-British Mission [Dummy]	0.036			-0.107^{**}		
	(0.051)			(0.039)		
British Mission [Count]		0.102^{***}			0.054^{***}	
		(0.020)			(0.015)	
Non-British Mission [Count]		−0.009			-0.036^{***}	
		(0.013)			(0.010)	
British Mission [Time]			0.617^{***}			0.463^{***}
			(0.124)			(0.079)
Non-British Mission [Time]			−0.078			-0.330^{***}
			(0.118)			(0.080)
Sample restriction	100km	25km	25km	100km	25km	25km
Individual controls	✓	✓	✓	✓	✓	✓
Colonial controls	✓	✓	✓	✓	✓	✓
Precolonial controls	✓	✓	✓	✓	✓	✓
AIC	45846.590	25861.613	25926.669	56971.427	34814.828	34787.413
BIC	46206.740	26200.740	26265.796	57331.577	35153.955	35126.540
Log Likelihood	−22882.295	−12889.807	−12922.334	−28444.714	−17366.414	−17352.707
Deviance	45764.590	25779.613	25844.669	56889.427	34732.828	34705.413
Num. obs.	48250	28894	28894	48250	28894	28894

Note: Logit models with colony and wave fixed-effects, and robust standard errors clustered by sampling location. Sample restricted to indicated distance from Protestant missions. Mission variables refer to Protestant stations within 25km radius from respondent and capture their presence, their number, respectively the time since the first establishment. See Table 1 for list of control variables. (*=.05, **=.01, ***=.001)

Table A3 Long-term Effects of Protestant Missions on Education Outcomes in British Africa (Ethnic Homelands FE)

	Primary Education			Years of Education		
	(1)	(2)	(3)	(4)	(5)	(6)
British Mission [Dummy]	0.039^{***}			0.544^{***}		
	(0.008)			(0.087)		
Non-British Mission [Dummy]	-0.002			-0.015		
	(0.008)			(0.089)		
British Mission [Count]		0.011^{***}			0.169^{***}	
		(0.003)			(0.035)	
Non-British Mission [Count]		-0.003			-0.060^{*}	
		(0.002)			(0.026)	
British Mission [Time]			0.066^{***}			0.761^{***}
			(0.018)			(0.212)
Non-British Mission [Time]			0.005			0.146
			(0.019)			(0.229)
Sample restriction	100km	25km	25km	100km	25km	25km
Individual Controls	✓	✓	✓	✓	✓	✓
Colonial controls	✓	✓	✓	✓	✓	✓
Precolonial controls	✓	✓	✓	✓	✓	✓
R^2	0.234	0.247	0.246	0.271	0.271	0.269
Adj. R^2	0.229	0.242	0.241	0.267	0.266	0.264
Num. obs.	56547	29288	29288	56547	29288	29288

Note: OLS with colony, wave, and ethnic homeland fixed-effects, and robust standard errors clustered by sampling location. Sample restricted to indicated distance from Protestant missions. Mission variables refer to Protestant stations within 25km radius from respondent and capture their presence, their number, respectively the time since the first establishment. Pre-colonial controls include altitude, terrain ruggedness, agricultural suitability, population density, malaria burden, access to waterways, and distances from the coast as well as Muslim centers; colonial controls include distances from the colonial capital, cities, railway access, and Catholic mission presence; individual controls include gender, age, and age squared (details in text). (*=.05, **=.01, ***=.001)

Table A4 Long-term Effects of Protestant Missions on Democratic Attitudes in British Africa (Ethnic Homelands FE)

	Support for Democracy			Support for Elections		
	(1)	(2)	(3)	(4)	(5)	(6)
British Mission [Dummy]	0.025**			0.052*		
	(0.008)			(0.023)		
Non-British Mission [Dummy]	−0.007			−0.012		
	(0.008)			(0.024)		
British Mission [Count]		0.007*			0.014	
		(0.003)			(0.011)	
Non-British Mission [Count]		−0.004			−0.007	
		(0.002)			(0.007)	
British Mission [Time]			0.051**			0.086
			(0.019)			(0.054)
Non-British Mission [Time]			−0.038			0.021
			(0.021)			(0.060)
Sample restriction	100km	25km	25km	100km	25km	25km
Individual Controls	✓	✓	✓	✓	✓	✓
Colonial controls	✓	✓	✓	✓	✓	✓
Precolonial controls	✓	✓	✓	✓	✓	✓
R^2	0.082	0.068	0.068	0.043	0.043	0.043
Adj. R^2	0.076	0.061	0.061	0.038	0.036	0.036
Num. obs.	56547	29288	29288	56547	29288	29288

Note: OLS with colony, wave, and ethnic homeland fixed-effects, and robust standard errors clustered by sampling location. Sample restricted to indicated distance from Protestant missions. Mission variables refer to Protestant stations within 25km radius from respondent and capture their presence, their number, respectively the time since the first establishment. See Table A3 for list of control variables. (*=.05, **=.01, ***=.001)

Table A5 Long-term Effects of Christian Missions in British Africa (excluding former German colonies)

	Primary Education	Years of Education	Support for Democracy	Support for Elections
	(1)	(2)	(3)	(4)
British Mission [Dummy]	0.041***	0.549***	0.036***	0.066**
	(0.008)	(0.092)	(0.008)	(0.021)
Non-British Mission [Dummy]	−0.001	0.056	−0.020*	−0.044*
	(0.009)	(0.097)	(0.008)	(0.022)
Sample restriction	100km	100km	100km	100km
Individual controls	✓	✓	✓	✓
Colonial controls	✓	✓	✓	✓
Precolonial controls	✓	✓	✓	✓
R^2	0.224	0.259	0.059	0.032
Adj. R^2	0.223	0.259	0.058	0.031
Num. obs.	42808	42808	42808	42808

Note: OLS with colony and wave fixed-effects, and robust standard errors clustered by sampling location. Sample restricted to 100km from Protestant missions; former German colonies excluded. Mission variables indicate the presence of a Protestant station within 25km radius from respondent. See Table 1 for list of control variables. (*=.05, **=.01, ***=.001)

Table A6 Long-term Effects of Christian Missions in British Africa (excluding Muslim areas)

	Primary Education	Years of Education	Support for Democracy	Support for Elections
	(1)	(2)	(3)	(4)
British Mission [Dummy]	0.036***	0.521***	0.035***	0.068**
	(0.008)	(0.089)	(0.008)	(0.021)
Non-British Mission [Dummy]	0.000	0.072	−0.019*	−0.043*
	(0.009)	(0.093)	(0.008)	(0.021)
Sample restriction	100km	100km	100km	100km
Individual controls	✓	✓	✓	✓
Colonial controls	✓	✓	✓	✓
Precolonial controls	✓	✓	✓	✓
R^2	0.224	0.263	0.057	0.030
Adj. R^2	0.223	0.262	0.056	0.029
Num. obs.	44915	44915	44915	44915

Note: OLS with colony and wave fixed-effects, and robust standard errors clustered by sampling location. Sample restricted to 100km from Protestant missions; observations within 100km of early Muslim centers excluded. Mission variables indicate the presence of a Protestant station within 25km radius from respondent. See Table 1 for list of control variables. (*=.05, **=.01, ***=.001)

Table A7 Long-term Effects of Christian Missions in British Africa (English vs Non-English Missions)

	Primary Education	Years of Education	Support for Democracy	Support for Elections
	(1)	(2)	(3)	(4)
English Mission [Dummy]	0.039***	0.523***	0.032***	0.093***
	(0.008)	(0.089)	(0.008)	(0.021)
Non-English Mission [Dummy]	0.007	0.145	−0.014	−0.028
	(0.007)	(0.083)	(0.008)	(0.020)
Sample restriction	100km	100km	100km	100km
Individual controls	✓	✓	✓	✓
Colonial controls	✓	✓	✓	✓
Precolonial controls	✓	✓	✓	✓
R^2	0.219	0.251	0.055	0.030
Adj. R^2	0.219	0.251	0.054	0.029
Num. obs.	48250	48250	48250	48250

Note: OLS with colony and wave fixed-effects, and robust standard errors clustered by sampling location. Sample restricted to 100km from Protestant missions. Mission variables indicate the presence of a Protestant station within 25km radius from respondent. See Table 1 for list of control variables. (*=.05, **=.01, ***=.001)

Table A8 Long-term Effects of Christian Missions on Contemporary Outcomes (Individual Controls Omitted)

	Primary Education	Years of Education	Support for Democracy	Support for Elections
	(1)	(2)	(3)	(4)
British Mission [Dummy]	0.040***	0.540***	0.035***	0.067**
	(0.008)	(0.093)	(0.008)	(0.021)
Non-British Mission [Dummy]	−0.000	0.045	−0.022**	−0.044*
	(0.009)	(0.099)	(0.008)	(0.020)
Sample restriction	100km	100km	100km	100km
Individual controls	-	-	-	-
Colonial controls	✓	✓	✓	✓
Precolonial controls	✓	✓	✓	✓
R^2	0.132	0.159	0.049	0.029
Adj. R^2	0.132	0.159	0.048	0.028
Num. obs.	48250	48250	48250	48250

Note: OLS with colony and wave fixed-effects, and robust standard errors clustered by sampling location. Sample restricted to indicated distance from Protestant missions. Mission variables indicate the presence of a Protestant station within 25km radius from respondent. See Table 1 for list of control variables. (*=.05, **=.01, ***=.001)

Table A9 Long-term Effects of Christian Missions in British Africa (without "bad controls")

	Primary Education	Years of Education	Support for Democracy	Support for Elections
	(1)	(2)	(3)	(4)
British Mission [Dummy]	0.039***	0.552***	0.033***	0.060**
	(0.009)	(0.097)	(0.008)	(0.021)
Non-British Mission [Dummy]	0.011	0.186	−0.013	−0.032
	(0.009)	(0.101)	(0.008)	(0.020)
Sample restriction	100km	100km	100km	100km
Individual controls				
Colonial controls				
Precolonial controls	✓	✓	✓	✓
R^2	0.126	0.149	0.047	0.028
Adj. R^2	0.126	0.149	0.046	0.027
Num. obs.	48250	48250	48250	48250

Note: OLS with colony and wave fixed-effects, and robust standard errors clustered by sampling location. Sample restricted to 100km from Protestant missions. Mission variables indicate the presence of a Protestant station within 25km radius from respondent. See Table 1 for list of control variables. (*=.05, **=.01, ***=.001)

Table A10 Long-term Effects of Christian Missions on Contemporary Outcomes (Additional Controls)

	Primary Education	Years of Education	Support for Democracy	Support for Elections
	(1)	(2)	(3)	(4)
British Mission [Dummy]	0.033**	0.453***	0.028**	0.068**
	(0.011)	(0.112)	(0.009)	(0.024)
Non-British Mission [Dummy]	−0.007	0.054	−0.028**	−0.047
	(0.011)	(0.115)	(0.010)	(0.025)
Sample restriction	100km	100km	100km	100km
Individual controls	✓	✓	✓	✓
Colonial controls	✓	✓	✓	✓
Precolonial controls	✓	✓	✓	✓
R^2	0.216	0.263	0.046	0.026
Adj. R^2	0.216	0.262	0.045	0.025
Num. obs.	33619	33619	33619	33619

Note: OLS with colony and wave fixed-effects, and robust standard errors clustered by sampling location. Sample restricted to indicated distance from Protestant missions. Mission variables indicate the presence of a Protestant station within 25km radius from respondent. See Table 1 for list of control variables; in addition, controls for cash crop cultivation and mining activities included. (*=.05, **=.01, ***=.001)

Table A11 Long-term Effects of Christian Missions (Dummy, Sensitivity 1)

	Primary Education	Years of Education	Support for Democracy	Support for Elections
	(1)	(2)	(3)	(4)
British Mission [Dummy]	0.038***	0.546***	0.035***	0.067**
	(0.008)	(0.088)	(0.008)	(0.022)
Non-British Mission [Dummy]	0.000	0.056	−0.018*	−0.039
	(0.008)	(0.092)	(0.008)	(0.022)
Sample restriction	50km	50km	50km	50km
Individual controls	✓	✓	✓	✓
Colonial controls	✓	✓	✓	✓
Precolonial controls	✓	✓	✓	✓
R^2	0.225	0.255	0.053	0.028
Adj. R^2	0.225	0.254	0.052	0.027
Num. obs.	37955	37955	37955	37955

Note: OLS with colony and wave fixed-effects, and robust standard errors clustered by sampling location. Sample restricted to indicated distance from Protestant missions. Mission variables indicate the presence of a Protestant station within 25km radius from respondent. See Table 1 for list of control variables. (*=.05, **=.01, ***=.001)

Table A12 Long-term Effects of Christian Missions (Dummy, Sensitivity 2)

	Primary Education	Years of Education	Support for Democracy	Support for Elections
	(1)	(2)	(3)	(4)
British Mission [Dummy]	0.039***	0.543***	0.032***	0.063**
	(0.008)	(0.087)	(0.008)	(0.021)
Non-British Mission [Dummy]	0.007	0.112	−0.020*	−0.041*
	(0.008)	(0.090)	(0.008)	(0.020)
Sample restriction	-	-	-	-
Individual controls	✓	✓	✓	✓
Colonial controls	✓	✓	✓	✓
Precolonial controls	✓	✓	✓	✓
R^2	0.207	0.239	0.059	0.029
Adj. R^2	0.206	0.239	0.059	0.028
Num. obs.	56547	56547	56547	56547

Note: OLS with colony and wave fixed-effects, and robust standard errors clustered by sampling location. Sample restricted to indicated distance from Protestant missions. Mission variables indicate the presence of a Protestant station within 25km radius from respondent. See Table 1 for list of control variables. (*=.05, **=.01, ***=.001)

Table A13 Long-term Effects of Christian Missions (Dummy, Sensitivity 3)

	Primary Education	Years of Education	Support for Democracy	Support for Elections
	(1)	(2)	(3)	(4)
British Mission [Dummy]	0.043***	0.462***	0.035***	0.049*
	(0.008)	(0.081)	(0.007)	(0.020)
Non-British Mission [Dummy]	0.004	0.063	−0.021**	−0.034
	(0.009)	(0.092)	(0.008)	(0.022)
Sample restriction	100km	100km	100km	100km
Individual controls	✓	✓	✓	✓
Colonial controls	✓	✓	✓	✓
Precolonial controls	✓	✓	✓	✓
R^2	0.216	0.246	0.056	0.029
Adj. R^2	0.216	0.246	0.055	0.029
Num. obs.	48250	48250	48250	48250

Note: OLS with colony and wave fixed-effects, and robust standard errors clustered by sampling location. Sample restricted to indicated distance from Protestant missions. Mission variables indicate the presence of a Protestant station within 50km radius from respondent. See Table 1 for list of control variables. (*=.05, **=.01, ***=.001)

Table A14 Long-term Effects of British Protestant Missions in British Colonies

	Primary Education	Years of Education	Support for Democracy	Support for Elections
	(1)	(2)	(3)	(4)
British Protestant Mission	0.058***	0.769***	0.023***	0.046*
	(0.007)	(0.083)	(0.007)	(0.020)
Sample restriction	100km	100km	100km	100km
Individual controls	✓	✓	✓	✓
Colonial controls	✓	✓	✓	✓
Precolonial controls	✓	✓	✓	✓
R^2	0.222	0.261	0.059	0.034
Adj. R^2	0.222	0.261	0.058	0.033
Num. obs.	40516	40516	40516	40516

Note: OLS with colony and wave fixed-effects, and robust standard errors clustered by sampling location. Sample restricted to indicated distance from Catholic missions. Mission variables indicate the presence of a Catholic station within 25km radius from respondent. See Table 1 for list of control variables. (*=.05, **=.01, ***=.001)

Table A15 Long-term Effects of Non-British Protestant Missions in British Colonies

	Primary Education	Years of Education	Support for Democracy	Support for Elections
	(1)	(2)	(3)	(4)
Non-British Protestant Mission	0.026**	0.357***	−0.010	−0.037
	(0.008)	(0.092)	(0.008)	(0.020)
Sample restriction	100km	100km	100km	100km
Individual controls	✓	✓	✓	✓
Colonial controls	✓	✓	✓	✓
Precolonial controls	✓	✓	✓	✓
R^2	0.229	0.250	0.052	0.031
Adj. R^2	0.228	0.250	0.051	0.030
Num. obs.	38139	38139	38139	38139

Note: OLS with colony and wave fixed-effects, and robust standard errors clustered by sampling location. Sample restricted to indicated distance from Catholic missions. Mission variables indicate the presence of a Catholic station within 25km radius from respondent. See Table 1 for list of control variables. (*=.05, **=.01, ***=.001)

Table A16 Long-term Effects of Protestant Missions with Catholic Competition in British Colonies

	Primary Education	Years of Education	Support for Democracy	Support for Elections
	(1)	(2)	(3)	(4)
British Protestant Mission	0.057***	0.758***	0.049***	0.085*
	(0.012)	(0.136)	(0.013)	(0.037)
Non-British Protestant Mission	−0.031*	−0.342*	−0.063***	−0.093*
	(0.015)	(0.169)	(0.014)	(0.043)
Sample restriction	100km	100km	100km	100km
Individual controls	✓	✓	✓	✓
Colonial controls	✓	✓	✓	✓
Precolonial controls	✓	✓	✓	✓
R^2	0.214	0.228	0.052	0.036
Adj. R^2	0.212	0.226	0.049	0.033
Num. obs.	16356	16356	16356	16356

Note: OLS with colony and wave fixed-effects, and robust standard errors clustered by sampling location. Sample restricted to locations with a Catholic mission and within indicated distance from Protestant missions. Mission variables indicate the presence of a Protestant station within 25km radius from respondent. See Table 1 for list of control variables. (*=.05, **=.01, ***=.001)

Table A17 Long-term Effects of Protestant Missions without Catholic Competition in British Colonies

	Primary Education	Years of Education	Support for Democracy	Support for Elections
	(1)	(2)	(3)	(4)
British Protestant Mission	0.022*	0.306**	0.026**	0.054*
	(0.009)	(0.098)	(0.010)	(0.026)
Non-British Protestant Mission	0.014	0.175	−0.016	−0.040
	(0.009)	(0.098)	(0.010)	(0.024)
Sample restriction	100km	100km	100km	100km
Individual controls	✓	✓	✓	✓
Colonial controls	✓	✓	✓	✓
Precolonial controls	✓	✓	✓	✓
R^2	0.223	0.259	0.063	0.029
Adj. R^2	0.222	0.258	0.062	0.028
Num. obs.	31894	31894	31894	31894

Note: OLS with colony and wave fixed-effects, and robust standard errors clustered by sampling location. Sample restricted to locations without a Catholic mission and within indicated distance from Protestant missions. Mission variables indicate the presence of a Protestant station within 25km radius from respondent. See Table 1 for list of control variables. (*=.05, **=.01, ***=.001)

Table A18 Long-term Effects of Religious Competition in British Colonies

	Primary Education	Years of Education	Support for Democracy	Support for Elections
	(1)	(2)	(3)	(4)
British Mission (after 1920)	0.030	0.175	0.034	0.009
	(0.029)	(0.324)	(0.023)	(0.070)
Non-British Mission (after 1920)	0.030	0.326	−0.019	−0.039
	(0.017)	(0.203)	(0.017)	(0.047)
British Mission (up to 1920)	0.029***	0.370***	0.028***	0.039
	(0.009)	(0.093)	(0.008)	(0.023)
Non-British Mission (up to 1920)	0.003	0.068	−0.020*	−0.038
	(0.009)	(0.094)	(0.008)	(0.021)
Sample restriction	100km	100km	100km	100km
Individual controls	✓	✓	✓	✓
Colonial controls	✓	✓	✓	✓
Precolonial controls	✓	✓	✓	✓
R^2	0.219	0.250	0.055	0.029
Adj. R^2	0.218	0.249	0.054	0.029
Num. obs.	48250	48250	48250	48250

Note: OLS with colony and wave fixed-effects, and robust standard errors clustered by sampling location. Sample restricted to indicated distance from Catholic missions. Mission variables indicate the presence of a Catholic station within 25km radius from respondent. See Table 1 for list of control variables. (*=.05, **=.01, ***=.001)

Table A19 Long-term Effects of Protestant Missions in British Colonies (High Democracy Sample)

	Primary Education	Years of Education	Support for Democracy	Support for Elections
	(1)	(2)	(3)	(4)
British Protestant Mission	0.052***	0.718***	0.026**	0.037
	(0.011)	(0.125)	(0.010)	(0.028)
Non-British Protestant Mission	0.010	0.151	−0.033**	−0.045
	(0.011)	(0.122)	(0.010)	(0.025)
Sample restriction	100km	100km	100km	100km
Individual controls	✓	✓	✓	✓
Colonial controls	✓	✓	✓	✓
Precolonial controls	✓	✓	✓	✓
R^2	0.201	0.235	0.054	0.036
Adj. R^2	0.199	0.234	0.053	0.035
Num. obs.	25957	25957	25957	25957

Note: OLS with colony and wave fixed-effects, and robust standard errors clustered by sampling location. Sample restricted to countries with high democracy scores in 1995 and indicated distance from Protestant missions. Mission variables indicate the presence of a Protestant station within 25km radius from respondent. See Table 1 for list of control variables. (*=.05, **=.01, ***=.001)

Table A20 Long-term Effects of Protestant Missions in British Colonies (Low Democracy Sample)

	Primary Education	Years of Education	Support for Democracy	Support for Elections
	(1)	(2)	(3)	(4)
British Protestant Mission	0.062***	0.756***	0.039***	0.065*
	(0.010)	(0.120)	(0.012)	(0.028)
Non-British Protestant Mission	0.003	0.139	0.004	0.004
	(0.010)	(0.129)	(0.013)	(0.031)
Sample restriction	100km	100km	100km	100km
Individual controls	✓	✓	✓	✓
Colonial controls	✓	✓	✓	✓
Precolonial controls	✓	✓	✓	✓
R^2	0.221	0.252	0.063	0.035
Adj. R^2	0.220	0.251	0.061	0.034
Num. obs.	19978	19978	19978	19978

Note: OLS with colony and wave fixed-effects, and robust standard errors clustered by sampling location. Sample restricted to countries with low democracy scores in 1995 and indicated distance from Protestant missions. Mission variables indicate the presence of a Protestant station within 25km radius from respondent. See Table 1 for list of control variables. (*=.05, **=.01, ***=.001)

Figure A1 Leave-One-Out Regressions for Primary Education

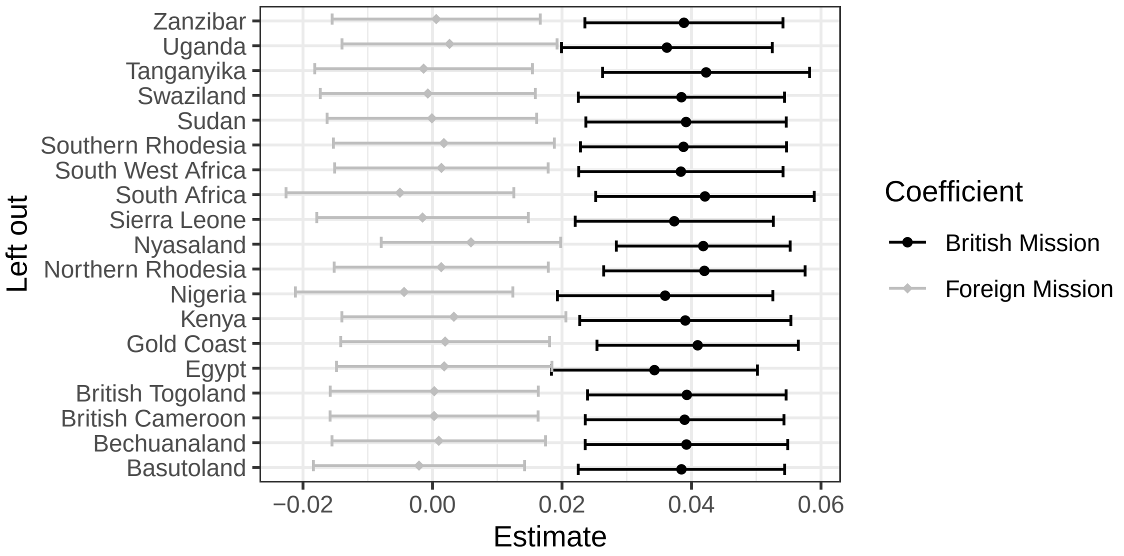

Notes: Re-estimations of model 1 in Table 1, with one colony removed from the sample.

Figure A2 Leave-One-Out Regressions for Education Years

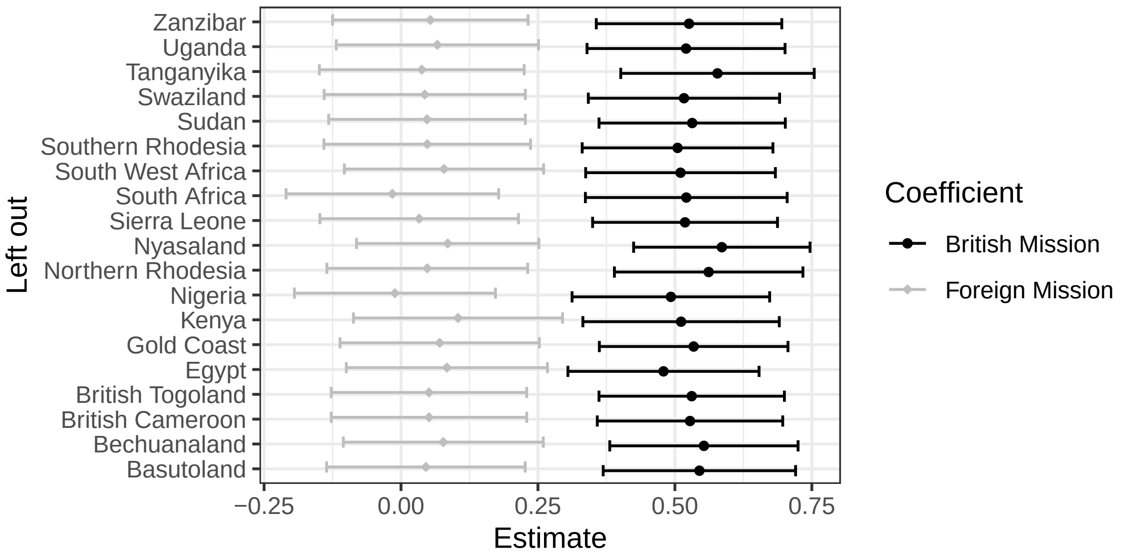

Notes: Re-estimations of model 4 in Table 1, with one colony removed from the sample.

Figure A3 Leave-One-Out Regressions for Support Democracy

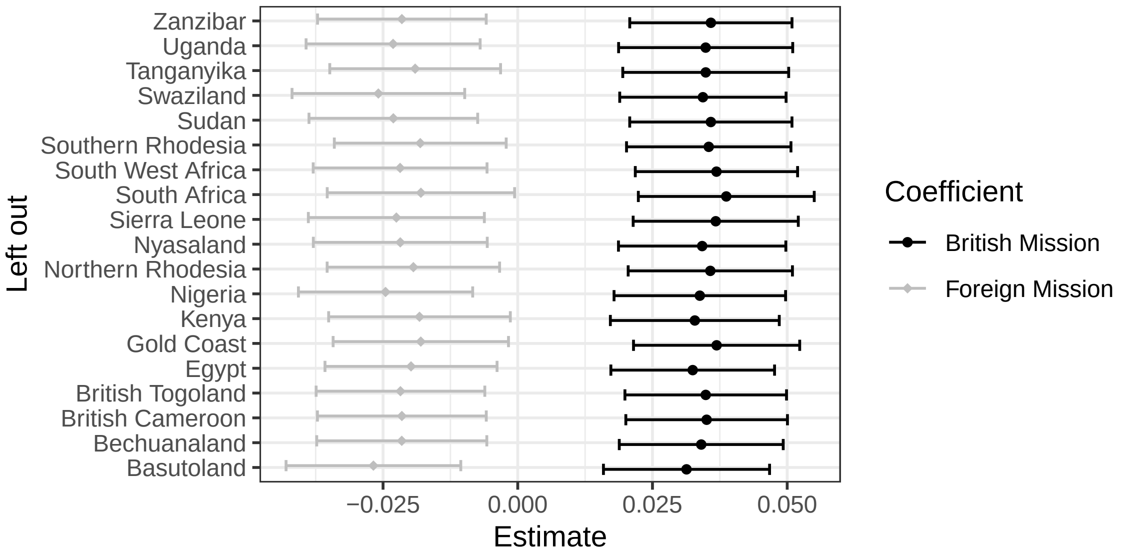

Notes: Re-estimations of model 1 in Table 2, with one colony removed from the sample.

Figure A4 Leave-One-Out Regressions for Support Elections

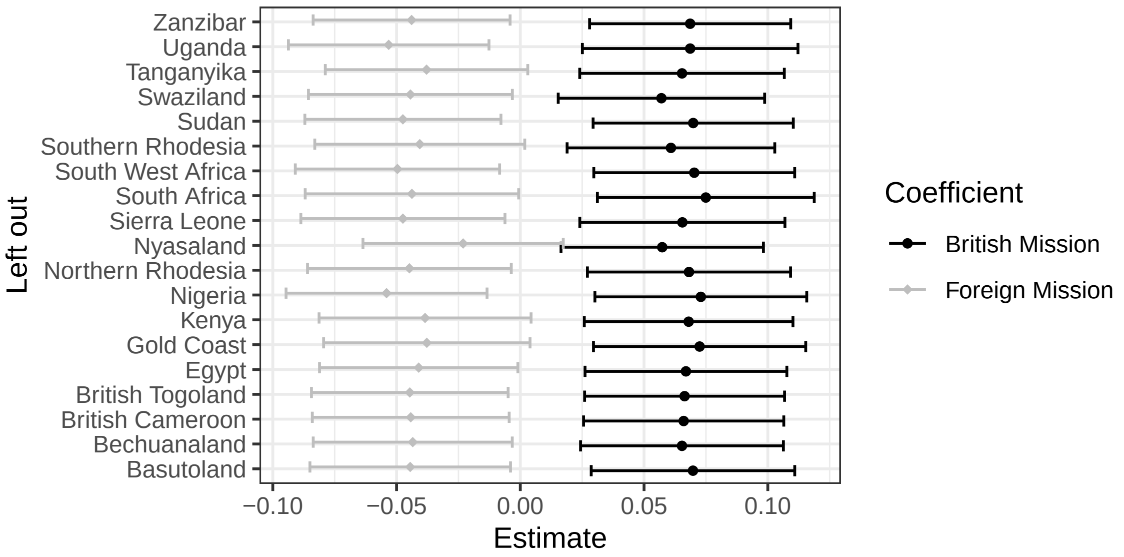

Notes: Re-estimations of model 4 in Table 2, with one colony removed from the sample.

Figure A5 Results of Mediation Models

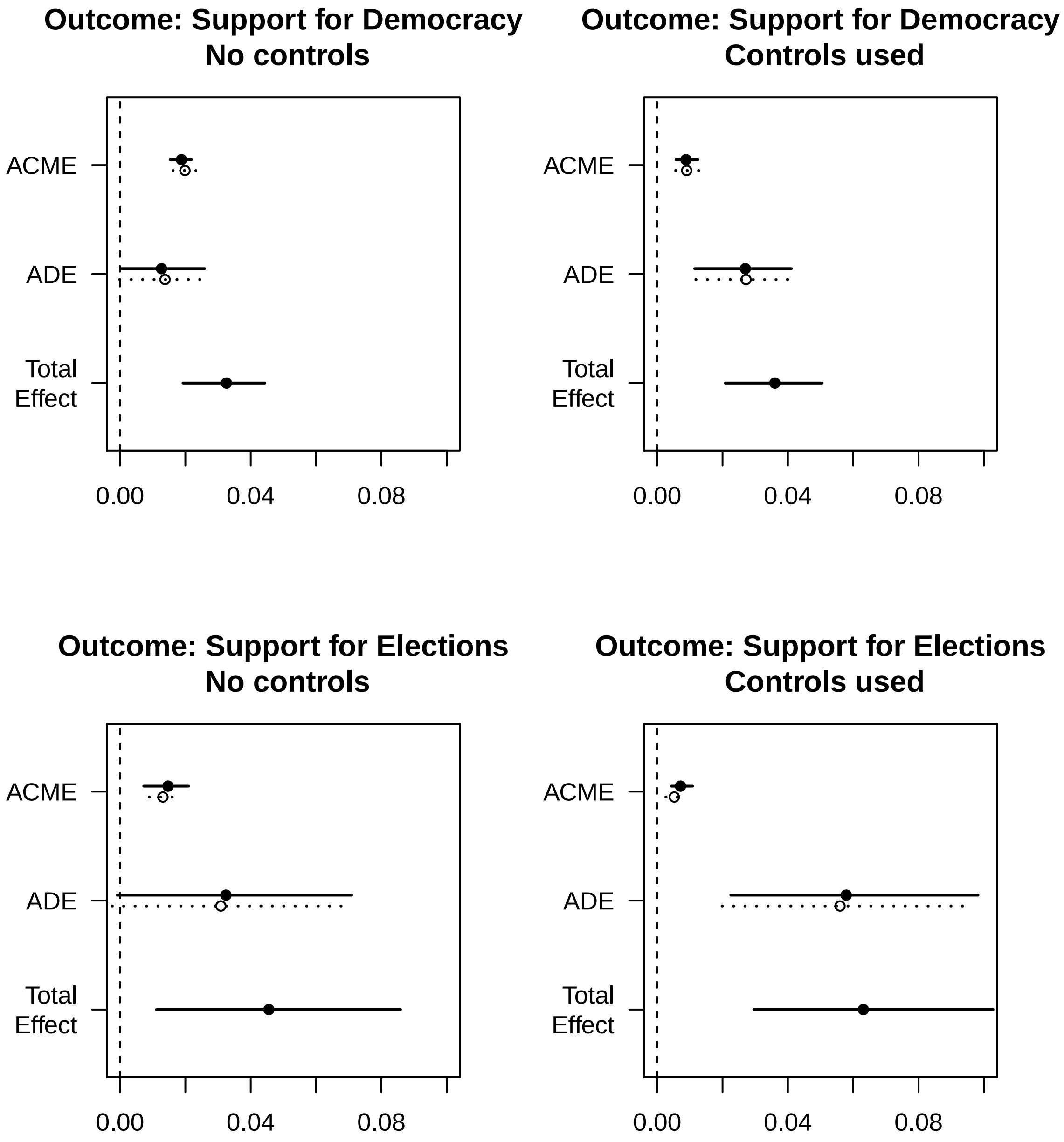

Notes: Mediation models with cluster-robust standard errors, with British Missions as treatment variable and Years of Education as mediator. ADE = Average Direct Effect; ACME = Average Causal Mediation Effect (Indirect Effect). For ADE and ACME, dashed lines refer to control group, solid lines refer to treatment group; averages reported in text. Lines capture 95%-confidence intervals. Control variables are listed in Table 1. Models estimated using R package `mediation`.

Figure A6 Catholic Mission Fields in Colonial Africa

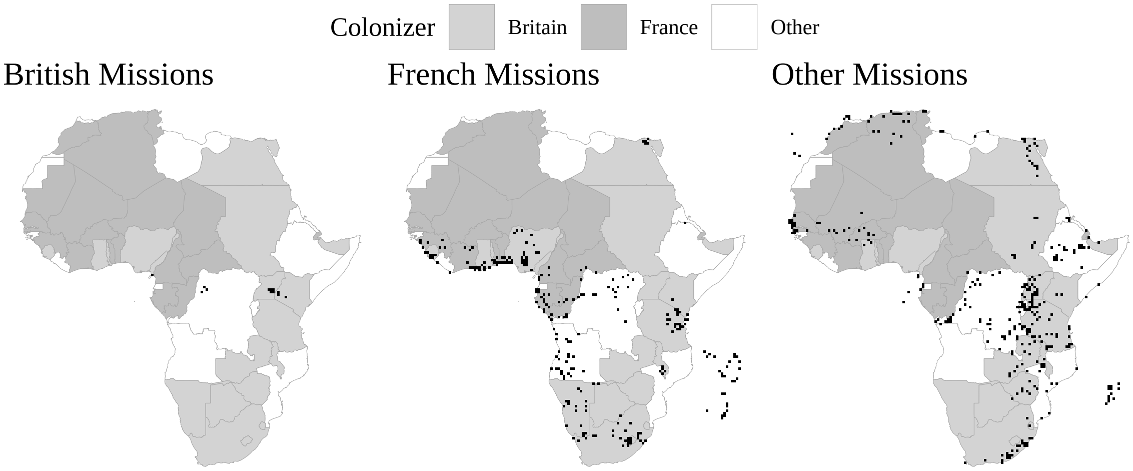

Notes: Grid cells (.5° × .5°) indicating the presence of a mission station. Based on the *Atlas Hierarchicus* (Streit, 1929).

References

Amasyalı, E. (2021). Missionary influence and nationalist reactions: The case of armenian ottomans. *Nationalities Papers*, *49*(3), 523–541. Publisher: Cambridge University Press.

Amasyalı, E. (2022). Protestant missionary education and the diffusion of women's education in ottoman turkey: A historical GIS analysis. *Social Science History*, 1–50. Publisher: Cambridge University Press. doi:10.1017/ssh.2021.39

Becker, B. (2021). The colonial struggle over polygamy: Consequences for educational expansion in sub-saharan africa. *Economic History of Developing Regions*, *37*(1). doi:10.1080/20780389.2021.1940946

Becker, B. (2022). The empire within: Longitudinal evidence on the expansion of christian missions in colonial africa. *Journal of Historical Political Economy*, *2*(2), 333–362. Publisher: Now Publishers, Inc. doi:10.1561/115.00000032

Fresh, A. (2018). Industrial revolution and political change: Evidence from the british isles. Vanderbilt University, Working Paper.

Global Agro-ecological Zones (GAEZ v3.0). (2011). Rome, Italy: FAO.

Hance, W. A., Kotschar, V., & Peterec, R. J. (1961). Source areas of export production in tropical africa. *Geographical Review*, *51*(4), 487–499. Publisher: [American Geographical Society, Wiley]. doi:10.2307/213104

Jedwab, R., Meier zu Selhausen, F., & Moradi, A. (2022). The economics of missionary expansion: Evidence from africa and implications for development. *Journal of Economic Growth*. doi:10.1007/s10887-022-09202-8

Jedwab, R., & Moradi, A. (2016). The permanent effects of transportation revolutions in poor countries: Evidence from africa. *Review of Economics and Statistics*. doi:10.1162/REST_a_00540

Klein Goldewijk, K., Beusen, A., & Janssen, P. (2010). Long-term dynamic modeling of global population and built-up area in a spatially explicit way: HYDE 3.1. *The Holocene*, *20*(4), 565–573. doi:10.1177/0959683609356587

Knutsen, C. H., Kotsadam, A., Olsen, E. H., & Wig, T. (2017). Mining and local corruption in africa. *American Journal of Political Science*, *61*(2), 320–334. doi:10.1111/ajps.12268

Lankina, T., & Getachew, L. (2013). Competitive religious entrepreneurs: Christian missionaries and female education in colonial and post-colonial india. *British Journal of Political Science*, *43*(1), 103–131. doi:10.1017/S0007123412000178

Lysenko, A., & Semashko, I. (1968). *Geography of malaria. a medico-geographic profile of an ancient disease*. Moscow.

Murdock, G. P. (1967). Ethnographic atlas: A summary. *Ethnology*, *6*(2), 109. doi:10.2307/3772751

Nunn, N. (2008). The long-term effects of africa's slave trades. *The Quarterly Journal of Economics*, *123*(1), 139–176. doi:10.1162/qjec.2008.123.1.139

Nunn, N., & Puga, D. (2012). Ruggedness: The blessing of bad geography in africa. *Review of Economics and Statistics*, *94*(1), 20–36. Publisher: MIT Press.

Pengl, Y. I., Roessler, P., & Rueda, V. (2021). Cash crops, print technologies, and the politicization of ethnicity in africa. *American Political Science Review*, 1–19. Publisher: Cambridge University Press. doi:10.1017/S0003055421000782

Roessler, P., Pengl, Y. I., Marty, R., Titlow, K. S., & van de Walle, N. (2020). *The cash crop revolution, colonialism and legacies of spatial inequality: Evidence from africa*. Centre for the Study of African Economies, University of Oxford.

Shariff, A. A., Norenzayan, A., & Henrich, J. (2011). The birth of high gods how the cultural evolution of supernatural policing influenced the emergence of complex, cooperative human societies. *Evolution, culture, and the human mind*. Publisher: Psychology Press.

Shaver, A., Carter, D. B., & Shawa, T. W. (2016). Terrain ruggedness and land cover: Improved data for most research designs: *Conflict Management and Peace Science*. Publisher: SAGE PublicationsSage UK: London, England. doi:10.1177/0738894216659843

Stasavage, D. (2014). Was weber right? the role of urban autonomy in europe's rise. *American Political Science Review*, *108*(2), 337–354. Publisher: Cambridge University Press.

Streit, K. (1929). *Atlas hierarchicus: Descriptio geographica et statistica sanctae romanae ecclesiae*. OCLC: 601406229. Paderborn: Typographia Bonifaciana.
